# Tim-3-Expressing CD4^+^ and CD8^+^ T Cells in Human Tuberculosis (TB) Exhibit Polarized Effector Memory Phenotypes and Stronger Anti-TB Effector Functions

**DOI:** 10.1371/journal.ppat.1002984

**Published:** 2012-11-08

**Authors:** Yueqin Qiu, Jianbo Chen, Hongying Liao, Yan Zhang, Hua Wang, Shaoyuan Li, Yanfen Luo, Danyun Fang, Guobao Li, Boping Zhou, Ling Shen, Crystal Y. Chen, Dan Huang, Jiye Cai, Kaiyuan Cao, Lifang Jiang, Gucheng Zeng, Zheng W. Chen

**Affiliations:** 1 Department of Microbiology, Zhongshan School of Medicine, Key Laboratory for Tropical Diseases Control of the Ministry of Education, Sun Yat-sen University, Guangzhou, China; 2 College of Life Sciences, Jinan University, Guangzhou, China; 3 Division of Infection and Immunity, Department for Clinical Microbiological Assays, Shenzhen Third People's Hospital, Shenzhen, China; 4 Department of Thoracic Surgery, The Third Affiliated Hospital of Sun Yat-sen University, Guangzhou, China; 5 Key Laboratory of Gene Engineering of the Ministry of Education, State Key Laboratory of Biocontrol, School of Life Sciences, Sun Yat-sen University, Guangzhou, China; 6 Department of Oral and Maxillofacial Surgery, Hospital of Stomotology, Guanghua School of Stomotology, Sun Yat-sen University, Guangzhou, China; 7 Department of Pulmonary Diseases, Shenzhen Third People's Hospital, Shenzhen, China; 8 Shenzhen Institute of Hepatology, Shenzhen Third People's Hospital, Shenzhen, China; 9 Harvard Medical School, Beth Israel Deaconess Medical Center, Boston, Massachusetts, United States of America; 10 Department of Microbiology and Immunology, Center for Primate Biomedical Research, University of Illinois College of Medicine, Chicago, Illinois, United States of America; Portland VA Medical Center/Oregon Health and Science University, United States of America

## Abstract

T-cell immune responses modulated by T-cell immunoglobulin and mucin domain-containing molecule 3 (Tim-3) during *Mycobacterium tuberculosis* (Mtb) infection in humans remain poorly understood. Here, we found that active TB patients exhibited increases in numbers of Tim-3-expressing CD4^+^ and CD8^+^ T cells, which preferentially displayed polarized effector memory phenotypes. Consistent with effector phenotypes, Tim-3^+^CD4^+^ and Tim-3^+^CD8^+^ T-cell subsets showed greater effector functions for producing Th1/Th22 cytokines and CTL effector molecules than Tim-3^−^ counterparts, and Tim-3-expressing T cells more apparently limited intracellular Mtb replication in macrophages. The increased effector functions for Tim-3-expressing T cells consisted with cellular activation signaling as Tim-3^+^CD4^+^ and Tim-3^+^CD8^+^ T-cell subsets expressed much higher levels of phosphorylated signaling molecules p38, stat3, stat5, and Erk1/2 than Tim-3- controls. Mechanistic experiments showed that siRNA silencing of Tim-3 or soluble Tim-3 treatment interfering with membrane Tim-3-ligand interaction reduced *de novo* production of IFN-γ and TNF-α by Tim-3-expressing T cells. Furthermore, stimulation of Tim-3 signaling pathways by antibody cross-linking of membrane Tim-3 augmented effector function of IFN-γ production by CD4^+^ and CD8^+^ T cells, suggesting that Tim-3 signaling helped to drive stronger effector functions in active TB patients. This study therefore uncovered a previously unknown mechanism for T-cell immune responses regulated by Tim-3, and findings may have implications for potential immune intervention in TB.

## Author Summary

Tuberculosis (TB), an infectious disease caused by *Mycobacterium tuberculosis* (Mtb) infection, remains a leading cause of morbidity and mortality worldwide. While CD4^+^ and CD8^+^ T-cell effector functions producing Th1 or cytotoxic cytokines are required to mount anti-mycobacterial immunity, insufficiency or failure to mount anti-mycobacterial effector functions by CD4^+^ and CD8^+^ T cells may lead to impaired immunity against TB. Therefore, it is important to elucidate functional characteristics and regulatory pathways for Mtb-specific CD4^+^ and CD8^+^ T cells during immune responses to Mtb infection. It was postulated that T-cell immunoglobulin and mucin domain-containing molecule 3 (Tim-3) might represent a T-cell exhaustion marker, and expression of Tim-3 on T cells may be linked to progressive loss of secretion of cytokines. Thus, Tim-3 expression on T cells might correlate with T-cell dysfunction and disease pathogenic events. However, T-cell immune responses modulated by Tim-3 in human TB disease remain poorly understood. Here, we found that up-regulation of Tim-3 expression in active human TB disease allows CD4^+^ and CD8^+^ T cells to mount stronger, but not impaired, anti-mycobacterium effector functions. This study therefore uncovers a previously unknown mechanism for T-cell immune responses regulated by Tim-3, and has an important implication for TB diagnostics and therapy.

## Introduction

Tuberculosis (TB), an infectious disease caused by *Mycobacterium tuberculosis* (Mtb) infection, remains a leading cause of morbidity and mortality worldwide [1]. CD4^+^ and CD8^+^ T cells may be important for host immune resistance to TB in humans [2], [3], [4], [5]. In mouse models of Mtb infection, IFN-γ and TNF-α produced by CD4^+^ and CD8^+^ T cells have been shown to be critical for immune control of Mtb infection [2], [4], [5]. In addition, CD8^+^ T cells may contribute to anti-Mtb immunity through releasing bactericidal molecule granulysin or cytotoxic molecules perforin and granzymes killing of Mtb-infected target cells [2], [4], [5], [6]. It is likely that CD4^+^ and CD8^+^ T-cell effector functions producing Th1 or cytotoxic cytokines are required to mount anti-mycobacterial immunity [2], [4], [5]. In this context, insufficiency or failure to mount anti-mycobacterial effector functions by CD4^+^ and CD8^+^ T cells may lead to impaired immunity against TB [2]. Therefore, it is important to elucidate functional characteristics and regulatory pathways for Mtb-specific CD4^+^ and CD8^+^ T cells during immune responses to Mtb infection.

T-cell immunoglobulin and mucin domain-containing molecule 3 (Tim-3) is a membrane protein initially identified as a negative regulator of Th1 immunity in mice [7], [8], [9]. It was postulated that Tim-3, like other members of T-cell inhibitory molecules such as programmed death 1 (PD-1) [10], [11], [12] and co-stimulatory receptor cytotoxic T-lymphocyte antigen-4 (CTLA-4) [13], might represent a T-cell exhaustion marker [12], [14], [15], [16], [17], [18], [19], [20]. A number of studies have suggested that abundant expression of Tim-3 on T cells may be linked to progressive loss of secretion of cytokines such as IL-2, TNF-α and IFN-γ in viral infections [15], [16], [19], [21], [22] or tumors [23], [24]. Thus, Tim-3 expression on T cells might correlate with T-cell dysfunction and disease pathogenic events. We have recently shown that Mtb infection can induce significant up-regulation of Tim-3 expression in macaques [25], suggesting that Tim-3 might be involved in host immune responses during Mtb infection in primates. However, it is not known whether Tim-3 expression or Tim-3 pathway plays a role in modulating T-cell immune responses during Mtb infection in macaques and humans. Elucidating how Tim-3 regulates anti-Mtb effector functions of CD4^+^ and CD8^+^ T cells in human TB will help to understand TB immunopathogenesis and have some implications for immune intervention in TB.

Given that Mtb-specific CD4^+^ and CD8^+^ T-cell responses are important for anti-mycobacterial immunity, and that Mtb infection drives up-regulation of Tim-3 capable of regulating T-cell effector functions, we hypothesize that Tim-3-expressing CD4^+^ and CD8^+^ T-cell subpopulations may play a role in modulating host immune responses during Mtb infection in humans. In the current study, we examined the expression of Tim-3 in TB patients in the context of functional characteristics of Mtb-specific CD4^+^ and CD8^+^ T cells. Surprisingly, we found that Tim-3-expressing CD4^+^ and CD8^+^ T cells in active TB patients exhibit polarized effector memory phenotypes and stronger but not impaired anti-mycobacterium effector functions. Our findings therefore may implicate a new paradigm for T-cell immune responses regulated by Tim-3 expression in human TB.

## Results

### Active TB patients exhibited up-regulation of expression of Tim-3 or Galectin-9 (Gal-9) and increases in numbers of Tim-3 or Gal-9-expressing CD4^+^ and CD8^+^ T cells

**Figure 1:**
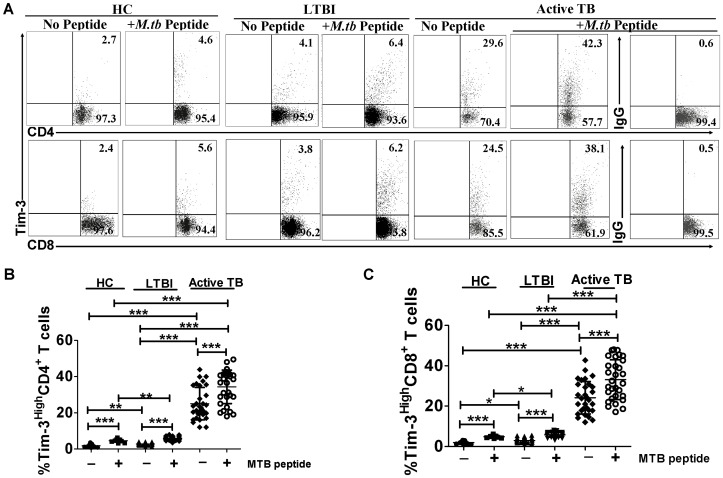
TB infection led to up-regulation of Tim-3 expression and increases in numbers of Tim-3-expressing CD4^+^ and CD8^+^ T cells. PBMC from 30 individuals with untreated active TB disease or 30 individuals with LTBI were stained directly or stimulated *ex vivo* first with pooled 15aa peptides overlapped by 12 spanning entire Mtb Ag85-b and ESAT-6 (termed Mtb peptide in figures), and then analyzed by polychromatic flow cytometry. (A) Representative flow cytometric dot plots showing Tim-3 expression in a healthy control (HC), a representative individual with untreated active TB disease, or a typical individual with LTBI. No Tim-3 expression was observed when we used isotype matched IgG to stain PBMC (see a representative panel on the right of Figure 1A). Values in the upper right quadrant indicate the percentages of Tim-3-expressing CD4^+^ and CD8^+^ T cells. Data were gated on CD3^+^CD4^+^ and CD3^+^CD8^+^, respectively. (B) and (C) are bar graph data showing that the percentages (%) of Tim-3 expression on CD4^+^ and CD8^+^ T cells from 30 patients with active TB disease are higher than those from 30 individuals with LTBI and 9 healthy control (HCs). Horizontal bars depict the mean percentage of Mtb-specific Tim-3 expression on CD4^+^ and CD8^+^ T cells. * *p<*0.05, ** *p<*0.01, *** *p<*0.001.

We previously demonstrated that Mtb infection induced up-regulation of Tim-3 expression in nonhuman primates [25]. As an initial step to characterize potential roles of Tim-3 expression in human TB, we performed *ex vivo* polychromatic flow cytometric analysis in 9 healthy controls (HCs), 30 subjects with latent TB infection (LTBI), and 30 untreated active TB patients (Clinical characteristics of the enrolled subjects with LTBI or active TB disease were shown in Supporting information, Table S1). Active TB was confirmed based on assessment of clinical syndromes, chest radiography, and acid-fast bacilli (AFB) staining in sputum, culture isolation of Mtb and PCR detection of Mtb genes [26]. Peripheral blood mononuclear cells (PBMC) isolated from healthy controls (HCs) or subjects with LTBI or active TB disease were stimulated *ex vivo* first with pooled Mtb Antigen 85-b (Ag85-b)/6 kDa early secretory antigenic target (ESAT-6) peptides (Mtb peptide pool) and then stained for Tim-3 and Gal-9, the only known ligand of Tim-3, or stained directly for Tim-3 or Gal-9 without peptide stimulation. In agreement with TB-driven up-regulation of Tim-3 and Gal-9 in Mtb-infected macaques [25], we found up-regulation of Tim-3 and Gal-9 and significant increases in numbers of Tim-3 or Gal-9-expressing CD4^+^ and CD8^+^ T cells in active TB patients when compared with HCs(Figure 1 and Supporting Information, Figure S1). Interestingly, Mtb peptide stimulation drove further increases in numbers of Tim-3 or Gal-9-expressing CD4^+^ and CD8^+^ T cells (Figure 1 and Supporting Information, Figure S1). The mean percentages of Tim-3-expressing CD4^+^ (or CD8^+^) T cells in PBMC of active TB disease and LTBI after Mtb peptide stimulation were increased approximately 9.3% and 3.0% (or 8.9% and 2.8%) more than those without Mtb peptide stimulation, respectively (Figure 1).These results suggested that active TB up-regulated Tim-3 and drove increases in numbers of Tim-3+CD4^+^ and CD8^+^ T cells.

### Tim-3-expressing CD4^+^ and CD8^+^ T cells in active TB patients preferentially displayed effector memory, but not central memory, phenotypes

To characterize phenotypic and functional profiles of Tim-3-expressing CD4^+^ and CD8^+^ T cells, we first examined whether Tim-3 was predominantly expressed in effector memory or central memory/naïve T-cell subsets in active TB. Blood CD4^+^ or CD8^+^ T cells from untreated active TB patients (n = 9) were co-stained for Tim-3 and naïve/memory markers in presence or absence of *ex vivo* antigenic stimulation by Mtb peptide pool, and then analyzed by polychromatic flow cytometry. Based on differential expression of CD45RA and CCR7 [27], [28], [29], 4 distinct T cell populations were classified as naïve T cells (T_naive_, CD45RA^+^CCR7^+^), central memory T cells (T_CM_, CD45RA^−^CCR7^+^), effector memory T cells (T_EM_, CD45RA^−^CCR7^−^), and RA^+^T_EM_ (T_EMRA_, also known as terminally differentiated; CD45RA^+^CCR7^−^) cells. We found that most of Tim-3-expressing CD4^+^ and CD8^+^ T cells freshly isolated from TB patients or stimulated *ex vivo* with Mtb peptide pool displayed CD45RA^−^CCR7^−^ T_EM_ phenotype, but not CD45RA^+^CCR7^+^ T_naïve_ or CD45RA^−^CCR7^+^ T_CM_ phenotype(Supporting Information, Figure S2), suggesting a polarization of effector memory phenotype for Tim-3-expressing CD4^+^ and CD8^+^ T cells in active TB patients.

Consistently, Tim-3-expressing CD4^+^ and CD8^+^ T cells expressed low levels of CD27 (Supporting Information, Figure S3A, B, C) and CD62L (Supporting Information, Figure S3D, E, F). In contrast to a lack of CCR7, CD62L, and CD27 expression, however, we found that Tim-3-expressing CD4^+^ and CD8^+^ T cells expressed high levels of CD127, another effector memory surrogate marker (Supporting Information, Figure S3G ,H, I). Furthermore, we determined whether Tim-3-expressing CD4^+^ and CD8^+^ T cells expressed CD27^−^CD45RA^−^ effector memory phenotype by co-staining Tim-3, CD27 and CD45RA on CD3^+^CD4^+^ and CD3^+^CD8^+^ T cells, respectively. We found that Tim-3-expressing CD4^+^ and CD8^+^ T cells preferentially displayed CD27^−^CD45RA^−^ effector memory phenotype during active TB disease (Supporting Information, Figure S4A, B, and C). Interestingly, we found that most of Tim-3-expressing CD4^+^ and CD8^+^ T cells during LTBI displayed CD27^−^CD45RA^−^effector memory phenotypes (Supporting Information, Figure S4 D, E, and F and data not shown). These results therefore demonstrated that Tim-3-expressing CD4^+^ and CD8^+^ T cells in active TB disease or LTBI preferentially displayed effector memory phenotypes.

### Tim-3^High^ CD4^+^ and CD8^+^ T-cell subsets exhibited greater effector functions for producing IFN-γ, TNF-α, IL-2 and IL-22 cytokines than their Tim-3^Low^ counterparts

Since Mtb-specific CD4^+^ and CD8^+^ T cells displaying effector memory phenotypes might be able to exert strong anti-mycobacterial effector functions [30], [31], we examined whether Mtb-driven Tim-3-expressing CD4^+^ and CD8^+^ T cells exhibited potent effector functions of cytokine production. We used two approaches to determine the relationship between Tim-3 expression and cytokine responses of CD4^+^ and CD8^+^ T cells: (i) PBMC from untreated active TB patients (n = 9) were stimulated *ex vivo* with Mtb peptides pool, and then stained for Tim-3 and anti-Mtb effector cytokines including IFN-γ, TNF-α, IL-2, and IL-22 and analyzed by polychromatic flow cytometry. This allowed us to evaluate correlation between TB-driven Tim-3 expression and Ag-stimulated cytokine responses of CD4^+^ and CD8^+^ T cells. (ii) PBMC from the same TB patients (n = 9) were directly stained for Tim-3 and the above cytokines without peptide stimulation as we recently described [32] to examine correlation between Tim-3 expression and an ability of Tim-3-expressing T cells to *de novo* produce cytokines. The specificity and utility of the direct intracellular cytokine staining approach has been validated during Mtb infection of macaques and humans as well as in the control settings [32], [33], [34].

**Figure 2:**
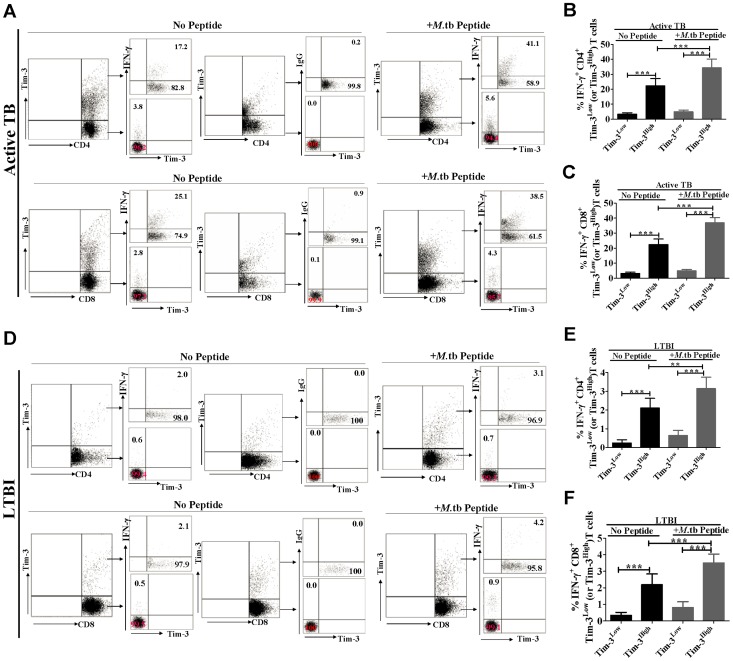
Tim-3^High^ CD4^+^ and CD8^+^ T-cell subsets showed greater effector functions for producing Mtb-specific IFN-γ and other cytokines (see Supporting Information, Figure S5, Figure S6) than their Tim-3^Low^ counterparts. PBMC derived from individuals with active TB disease (n = 9) or with LTBI (n = 9) were cultured *ex vivo* in presence or absence of pooled Ag85-b/ESAT-6 peptides, stained for fluorochrome-conjugated mAbs and analyzed by polychromatic flow cytometry. To examine the association between the IFN-γ response and Tim-3 expression, we used a two-tiered gating system for analyzing IFN-γ response by Tim-3-expressing T cells (Tim-3^High^) and Tim-3-negative cells (Tim-3^Low^) subpopulations. (A) shows representative flow cytometric dot plots derived from an untreated active TB patient, indicating percentages of IFN-γ-producing cells in Tim-3^High^CD4^+^ (or CD8^+^) T-cell subset versus Tim-3^Low^CD4^+^ (or CD8^+^) T-cell subset under the conditions with or without *ex vivo* stimulation with pooled Ag85B/ESAT-6 peptide. No significant intracellular staining of IFN-γ and other cytokines was seen when using isotype Ig control (see a representative panel in Figure 2A). (D) Similar flow cytometric dot plots show numbers of IFN-γ-producing cells in Tim-3^High^CD4^+^ (or CD8^+^) T cells versus Tim-3^Low^CD4^+^ (or CD8^+^) T cells in an individual with LTBI. (B) shows bar graph data from individuals with active TB disease (n = 9) and demonstrates that percentages of IFN-γ^+^ T cells within Tim-3^High^CD4^+^ T-cell subsets are much greater than those within Tim-3^Low^CD4^+^T cell subsets. (C) is similar to (B), except that data are IFN-γ^+^ T cells within Tim-3^High^CD8^+^ T-cell subsets. (E) are bar graph data from individuals with LTBI (n = 9) showing that percentages of IFN-γ^+^ T cells within Tim-3^High^CD4^+^ T-cell subsets are much greater than those within Tim-3^Low^CD4^+^T cell subset. (F) is similar to (E), except that data are IFN-γ^+^ T cells within Tim-3^High^ (or Tim-3^Low^)CD8^+^ T-cell subsets. Shown are data from at least three independent experiments. ** *p<*0.01, *** *p<*0.001. Error bars represent SD. Note that frequencies of cytokine-producing cells are expressed within Tim-3^+^ or Tim-3^−^IFN-γ^+^CD4^+^ or CD8^+^ T subsets, not total CD4^+^ or CD8^+^ T-cell population. Numbers of IFN-γ-producing cells within peptide-stimulated and unstimulated Tim-3^+^CD4^+^(or CD8^+^) T-cell subsets in active TB patients are significantly greater than those in healthy subjects with LTBI(*p*<0.001).

Interestingly, in the absence of Mtb peptide stimulation, approximately 18–21% of Tim-3^High^CD4^+^ T-cell subset from active TB patients were able to produce IFN-γ(Figure 2A, B), TNF-α (Supporting Information, Figure S5A, B), IL-2(Supporting Information, Figure S5C, D), and IL-22(Supporting Information, Figure S5E, F). In the presence of Mtb peptide stimulation, 30–34% of Tim-3^High^CD4^+^ T-cell subset could produce those cytokines(Figure 2A,B, Figure S5C,D,E,F). In contrast, Tim-3^Low^CD4+ T-cell subset did not produce appreciable levels of those cytokines regardless of peptide stimulation(Figure 2A,2B, and Supporting Information, Figure S5A,3B, Figure S5C,D, Figure S5E,F). Similarly, high percentages of Tim-3^High^CD8+ T-cell subset, but not Tim-3^Low^CD8^+^ T-cell subset, from these subjects with active TB disease were able to produce IFN-γ^+^(Figure 2A,C), TNF-α^+^(Supporting Information, Figure S6A, B), IL-2^+^(Supporting Information, Figure S6C, D) in the presence or absence of *ex vivo* stimulation with Mtb peptide pool. Interestingly, only ≤2.2% and ≤3.5% cells in Tim-3^High^CD4^+^(or CD8^+^) T cells from subjects with LTBI were able to produce cytokines in the absence and presence of Mtb peptide, respectively (Figure 2D,E,F, Figure S5, and Figure S6), implicating that TB inflammation or disease course contributed to large increase in Tim-3^High^ T effector cells actively producing cytokines. Notably, Mtb peptide stimulation led to ∼10%(∼12%) and ∼1%(∼1.2%) more increases in cytokine-producing cells in Tim-3^High^CD4^+^(or CD8^+^) T-cell subset in active TB and LTBI, respectively, when compared to the culture without peptide stimulation (Figure 2, Figure S5, Figure S6), These results suggested that the at least some of Tim-3^+^ T-cell effector cells were Mtb-specific. Thus, these results demonstrated that consistent with effector phenotypes, Tim-3^High^ CD4^+^ and CD8^+^ T-cell subsets exhibited greater effector functions for producing TB-driven IFN-γ, TNF-α, IL-2 and IL-22 cytokines than their Tim-3^Low^ counterparts.

### Tim-3^High^ CD4^+^ and CD8^+^ T-cell subsets demonstrated greater effector functions of cytotoxic molecule production and degranulation than their Tim-3^Low^ counterparts

**Figure 3:**
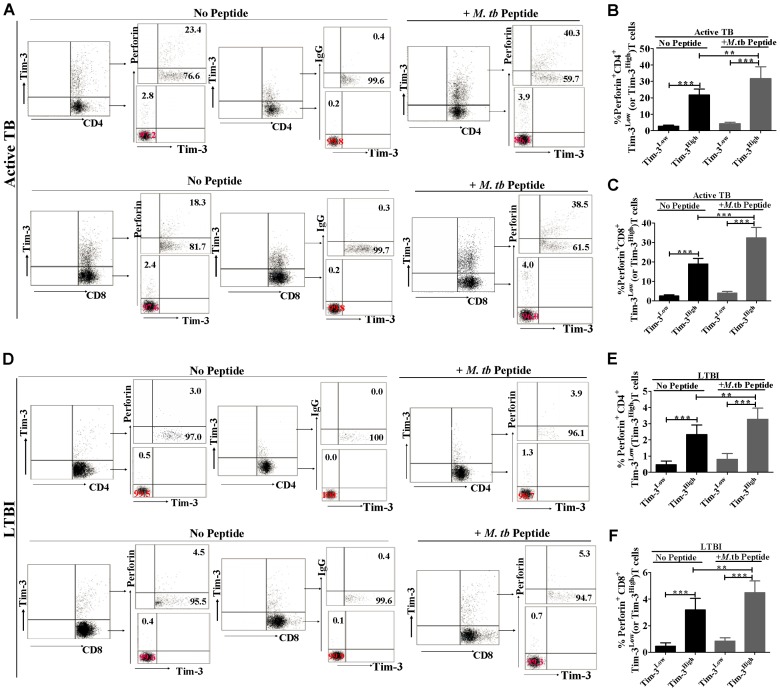
Tim-3^High^ CD4^+^ and CD8^+^ T-cell subsets also had greater effector functions of cytotoxic molecule production than their Tim-3^Low^ counterparts. PBMC from individuals with active TB disease (n = 9) or with LTBI (n = 9) were cultured in presence or absence of pooled Ag85-b/ESAT-6 peptides, stained with fluorochrome-conjugated mAbs, and analyzed by polychromatic flow cytometry. (A) shows representative flow cytometric dot plots derived from an untreated active TB patient, indicating percentages of perforin-producing cells in Tim-3^High^CD4^+^ (or CD8^+^) T-cell subset versus Tim-3^Low^CD4^+^ (or CD8^+^) T-cell subset under the conditions with or without *ex vivo* stimulation with pooled Ag85B/ESAT-6 peptide. (D) Similar flow cytometric dot plots show numbers of perforin-producing cells in Tim-3^High^CD4^+^ (or CD8^+^) T cells versus Tim-3^Low^CD4^+^ (or CD8^+^) T cells in an individual with LTBI. (B) shows bar graph data from individuals with active TB disease (n = 9) and demonstrates that percentages of perforin^+^ T cells within Tim-3^High^CD4^+^ T-cell subsets are much greater than those within Tim-3^Low^CD4^+^T cell subsets. (C) is similar to (B), except that data are perforin^+^ T cells within Tim-3^High^ (or Tim-3^Low^)CD8^+^ T-cell subsets. (E) are bar graph data from individuals with LTBI (n = 9) showing that percentages of perforin^+^ T cells within Tim-3^High^CD4^+^ T-cell subsets are much greater than those within Tim-3^Low^CD4^+^T cell subsets. (F) is similar to (E), except that data are perforin^+^ T cells within Tim-3^High^(or Tim-3^Low^)CD8^+^ T-cell subsets. Shown are data from at least three independent experiments. ** *p<*0.01, *** *p<*0.001. Error bars represent SD. Numbers of perforin-producing cells within peptide-stimulated and unstimulated Tim-3^+^CD4^+^(or CD8^+^) T-cell subsets in active TB patients are significantly greater than those in healthy subjects with LTBI (*p*<0.001).

To further characterize the Tim-3 expression and T-cell effector function, Tim-3^High^ T-cell subsets were assessed for the ability to produce cytotoxic effector molecuels perforin and granzyme B in comparisons with their Tim-3^Low^ counterparts. Two above approaches were similarly used in active TB patients (n = 9) and individuals with LTBI (n = 9). We found that much higher percentages of Tim-3^High^ CD4^+^ and CD8^+^ T-cell subsets produced perforin (Figure 3) and granzyme B (Supporting Information, Figure S7) than their Tim-3^Low^ controls regardless of presence or absence of Mtb peptide stimulation in either active TB disease or LTBI. Similarly, we observed that, compared with those without peptide stimulation, Tim-3^High^CD4^+^ and Tim-3^High^CD8^+^T-cell subsets showed stronger T-cell effector function of perforin and granzyme B production upon Mtb peptide stimulation (Figure 3 and Supporting information, Figure S7). To evaluate CTL-related degranulation capacity, Tim-3^High^ T-cell subsets were assessed for CD107a expression on cell surface, as CD107a, a lysosome-associated membrane glycoprotein, is expressed on cell surface following release of the cytotoxic granule contents, and is usually considered a hallmark for the degranulation capacity of CTL [20]. When compared with Tim-3^Low^ controls, Tim-3^High^ subsets of CD4^+^ and CD8^+^ T cells from individuals with either active TB disease (n = 9) expressed much higher levels of surface CD107a(Supporting Information, Figure S8). Similar higher levels of CD107 expression in Tim-3^High^CD4^+^ and Tim-3^High^CD8^+^ T-cell subsets were also observed in PBMC from individuals with LTBI (data not shown). Thus, Tim-3^High^CD4^+^ and Tim-3^High^CD8^+^ T-cell subsets in active TB disease and LTBI demonstrated greater effector functions of production of Mtb-driven cytotoxic molecules and degranulation than their Tim-3^Low^ counterparts.

### Tim-3 silencing by siRNA or interfering with Tim-3 signaling by addition of soluble Tim-3 (s-Tim-3) led to decreases in *de novo* production of IFN-γ and TNF-α by Tim-3-expressing T cells

**Figure 4:**
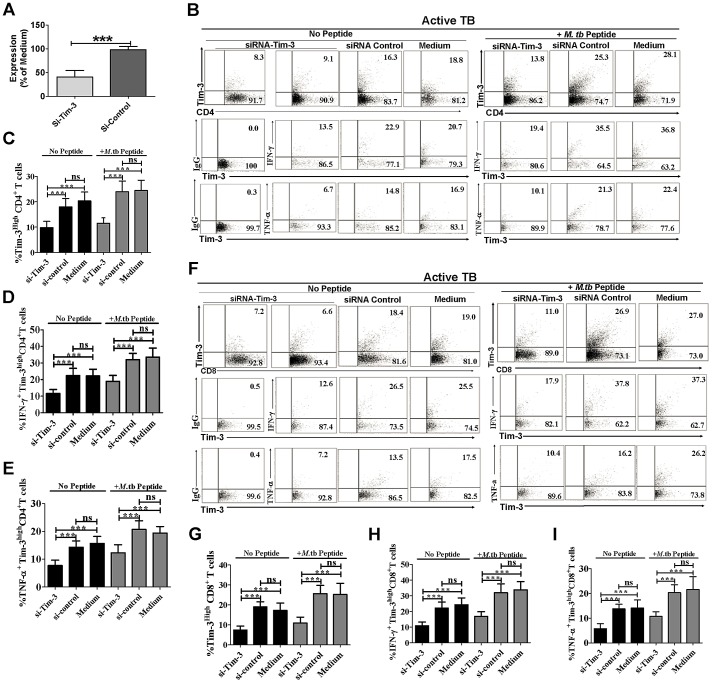
Tim-3 knockdown by siRNA led to decreases in *de novo* production of IFN-γ and TNF-α by CD4^+^ and CD8^+^ T cells. PBMC isolated from untreated active TB patients (n = 9) were transiently transfected with Tim-3-targeting (si-Tim-3) or control siRNA (si-control). Cells were then stimulated with or without pooled Ag85-b/ESAT-6 peptides for 6 days, stained by ICS with fluorochrome-conjugated mAbs and evaluated for the effects of Tim-3 silencing on T-cell effector functions using polychromatic flow cytometry. (A) Real-time PCR analysis of the expression of *Tim-3* in PBMC from Mtb-infected subjects (n = 9) at 48 hrs after transient transfection of Tim-3-targeted siRNA(si-Tim-3) and non-targeted siRNA(si-control), with data presented as values relative to expression in PBMC treated with Lipofectamine medium. (B) Typical flow cytometric dot plots and (C) summary bar graphic data show that Tim-3-specific siRNA (si-Tim-3), but not non-targeted siRNA(si-control) or Lipofectamine medium, specifically reduces the *de novo* or Mtb-driven expression of Tim-3 in CD4^+^ and CD8^+^ T cells. Values in upper right quadrant of flow cytometric dot plots indicate the percentages of Tim-3^High^ cells. (B) Flow cytometric dot plots and (D, E) summary bar graphic data show that Tim-3-targeted siRNA (si-Tim-3), but not non-targeted siRNA(si-control) or Lipofectamine medium, significantly reduces the percentages of IFN-γ- and TNF-α-expressing T cells in Tim-3^High^CD3^+^CD4^+^ T cells. Dot plots in (B) were gated on Tim-3^High^CD3^+^CD4^+^ T cells. (F), (G), (H), and (I) show that similar results in Tim-3^High^CD3^+^CD8^+^T cells. Dot plots in (F) were gated on Tim-3^High^CD3^+^CD8^+^ T cells. Values in each of flow cytometric dot plots are percentages of IFN-γ- or TNF-α-expressing T cells in Tim-3^High^CD3^+^CD4(or CD8)^+^ T cells. Data are from at least three independent experiments. Error bars represent SD. *** *p<*0.001, NS, no statistical significance.

To further explore the relationship between Tim-3 expression and effector functions of Tim-3-expressing T cells, we made use of siRNA targeting Tim-3 to knockdown the expression of Tim-3, and evaluated the effects of Tim-3 silencing on production of IFN-γ and TNF-α. siRNA targeting Tim-3(si-Tim-3), but not nontargeting siRNA (si-Control), could significantly knockdown *Tim-3*, as determined by RT-PCR (Figure 4A) and flow cytometry (Figure 4B, C, F, G). More importantly, silencing of Tim-3 by siRNA in CD4^+^ and CD8^+^ T cells led to significant decreases in production of IFN-γ and TNF-α (Figure 4B, D, E, F, H, I), when compared to the controls. These results suggested that Tim-3 expression in active TB was linked to potent IFN-γ and TNF-α responses of CD4^+^ and CD8^+^ T cells.

**Figure 5:**
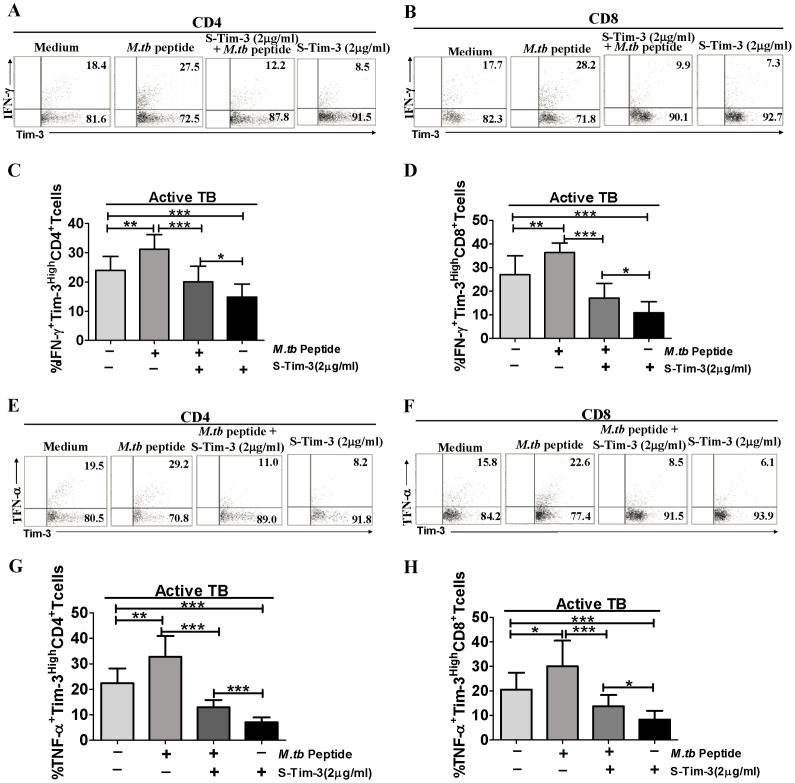
Adding a low concentration of soluble Tim-3 (s-Tim-3) to PBMC cultures reduced effector functions of IFN-γ and TNF-α production by Tim-3-expressing CD4^+^ and CD8^+^ T cells. PBMC derived from untreated active TB patients (n = 9) were incubated with soluble form of Tim-3 (s-Tim-3) in a concentration of 2 µg/ml in presence or absence of pooled Mtb Ag85-b/ESAT-6 peptides for 6 days. Cells were then stained using the ICS protocol, and analyzed by flow cytometry. (A) and (E) show representative flow cytometric data demonstrating the effects of Tim-3 ligand competition on Mtb-driven IFN-γ and TNF-α responses of Tim-3^High^CD4^+^ T cells. (C) and (G) show bar graphic data demonstrating that ligand competition by adding 2 µg/ml of soluble Tim-3 (s-Tim-3) significantly inhibits Mtb-driven IFN-γ and TNF-α production by CD4^+^ T cells. (B), (D), (F), and (H) show similar results were also observed in Tim-3^High^CD8^+^ T cells. Data are from at least two independent experiments. Error bars represent SD. *p<0.05, ** *p<*0.01 *** *p<*0.001.

On the other hand, we used Tim-3 ligand competition approach [19] to inhibit Tim-3 signaling pathways and examine the role of Tim-3 signaling in effector function of IFN-γ and TNF-α production by CD4^+^ and CD8^+^ T cells. PBMC from active TB patients (n = 9) were cultured in the presence of low concentration of soluble Tim-3 [termed s-Tim-3, i.e. Tim-3-Ig [7]] under the conditions with or without Mtb peptide pool stimulation. Interestingly, addition of low concentration of s-Tim-3 (2 µg/ml) for interfering with membrane Tim-3-Tim-3 ligand interaction significantly reduced the ability of Tim-3^High^ T-cell subsets to produce IFN-γ and TNF-α cytokines (Figure 5). These results suggested that soluble Tim-3 interfering with membrane Tim-3-ligand interaction could impact Tim-3 signaling pathways leading to decreases in effector function of IFN-γ and TNF-α production by Tim-3^High^ CD4^+^ and CD8^+^ T cells. However, cytokine-producing T cells in the culture of s-Tim-3 plus Mtb peptide were significantly higher than those of s-Tim-3 alone, suggesting again that some of cytokine-producing Tim-3^+^CD4^+^ or CD8^+^ T cells might be specific for Mtb antigens. The data also supported the notion that Tim-3 expression in active TB helped to drive stronger anti-microbial effector functions of CD4^+^ and CD8^+^ T cells.

### Stimulation of Tim-3 signaling pathways by Ab cross-linking of membrane Tim-3 augmented effector function of IFN-γ production by CD4^+^ and CD8^+^ T cells

We then examined whether enhancing of Tim-3 signaling could augment effector function of IFN-γ production by CD4^+^ and CD8^+^ T cells. Because anti-Tim-3 mAb could cross-link membrane Tim-3 and enhance Tim-3 signaling [35], [36], we used this approach to enhance Tim-3 signaling. PBMC from active TB patients (n = 9) were incubated with anti-Tim-3 mAb or isotype control Ab in presence or absence of Mtb peptide pool, and then assessed for potential effects of enhancing Tim-3 signal on IFN-γ production by CD4^+^ and CD8^+^ T cells. Interestingly, addition of anti-Tim-3 mAb, but not control Ab, to PBMC cultures resulted in significant increases in effector function of IFN-γ production by Tim-3^High^ CD4^+^ and CD8^+^ T-cell subsets (Supporting Information, Figure S9). Similar enhancement of T-cell effector functions by cross-linking Tim-3 on T-cell surface was also observed in PBMC of HCs and subjects with LTBI (data not shown). These results provided additional support for the hypothesis that Tim-3 signaling in CD4^+^ and CD8^+^ T cells helped to promote effector function of Th1 cytokine production.

### Tim-3^High^ T cells in active TB patients mounted stronger anti-Mtb effector function limiting intracellular Mtb replication in macrophages (MΦs)

**Figure 6:**
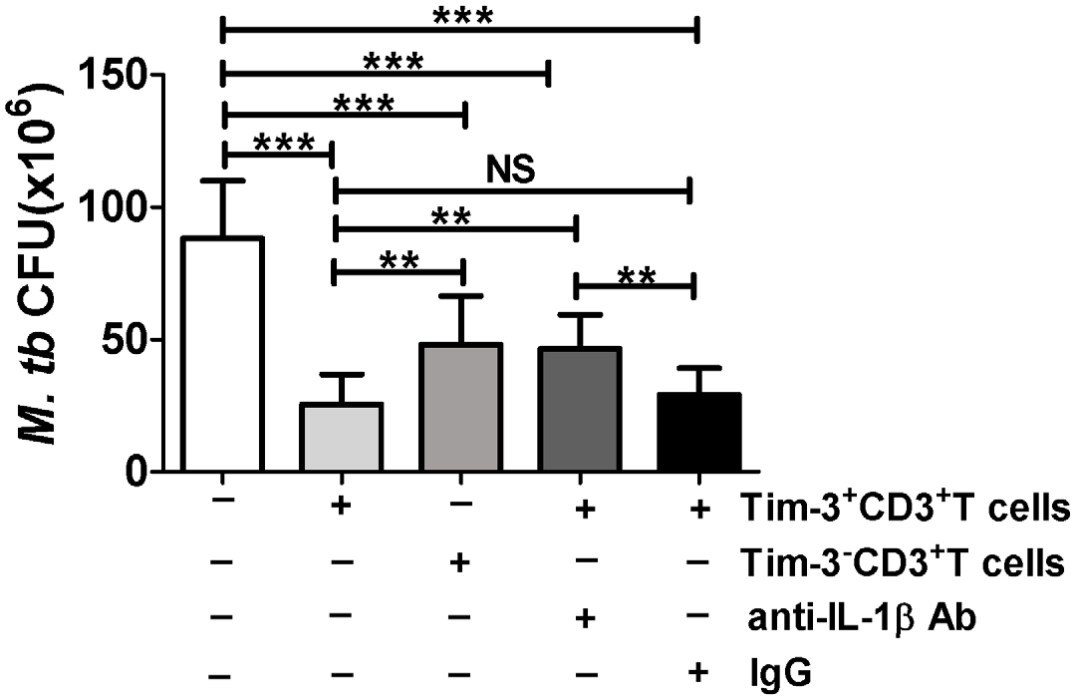
Purified Tim-3^+^CD3^+^T cells more effectively restricted Mtb replication in MΦs than Tim-3^−^CD3^+^T cells. PBMC were derived from untreated active TB patients (n = 9). Monocytes-derived MΦs were infected with Mtb overnight, and extracellular Mtb were then removed by extensive PBS wash. Autologous Tim-3^+^CD3^+^T cells and Tim-3^−^CD3^+^T cells were purified by magnetic beads and co-cultured with Mtb-infected MΦs for 4 days in presence or absence of anti-IL-1β Ab and isotype control IgG. Cultured cells containing Mtb-infected cells were then lysed, and Mtb CFUs were examined (see details in Materials and Methods). CFU numbers were expressed as per 10^6^ MΦs. Data are from at least three independent experiments. Error bars represent SD. *** *p<*0.001,** *p<*0.01, NS, no statistical significance.

While Tim-3 expression could regulate immune status of MΦs [35], our results implicated that Tim-3 expression enhanced T-cell effector functions for producing anti-microbial cytokines and cytotoxic molecules. Because it has also been suggested that IL-1β might contribute to the Tim-3-mediated inhibition of intracellular Mtb growth [37], we then asked whether Tim-3-expressing T cells could limit intracellular Mtb replication in MΦs, and whether IL-1β played a role in Tim-3^+^ T cell-induced anti-Mtb effector function. To address these questions, Tim-3-expressing CD3**^+^** T cells (Tim-3**^+^**CD3**^+^** T cells) were isolated from PBMCs of active TB patients(n = 9) using magnetic beads techniques, as we described [34], and co-cultured with Mtb-infected autologous MΦs in presence or absence of anti-IL-1β Ab or isotype control Ab. The co-cultured cells were then lysed, and lysate was measured for Mtb CFU counts on plates as we previously described [34]. Tim-3**^−^** CD3**^+^** T cells served as control. Interestingly, Tim-3-expressing T cells more apparently limited intracellular Mtb growth than Tim-3^−^ CD3^+^ T cells (Figure 6). Furthermore, treatment with anti-IL-1β Ab, but not isotype control Ab, could reverse the Tim-3^+^CD3^+^ T cell-mediated inhibition of intracellular Mtb growth, suggesting that IL-1β might contribute to the limitation of intracellular Mtb growth mediated by Tim-3^+^ CD3^+^ T cells. Thus, Tim-3-expressing T cells in active TB patients appeared to mount stronger anti-Mtb effector function limiting intracellular Mtb replication in cultured MΦs.

### Tim-3^High^ CD4^+^ and CD8^+^ T-cell subsets in active TB patients expressed higher levels of phosphorylated signaling molecules p38, stat3, stat5, and Erk1/2

**Figure 7:**
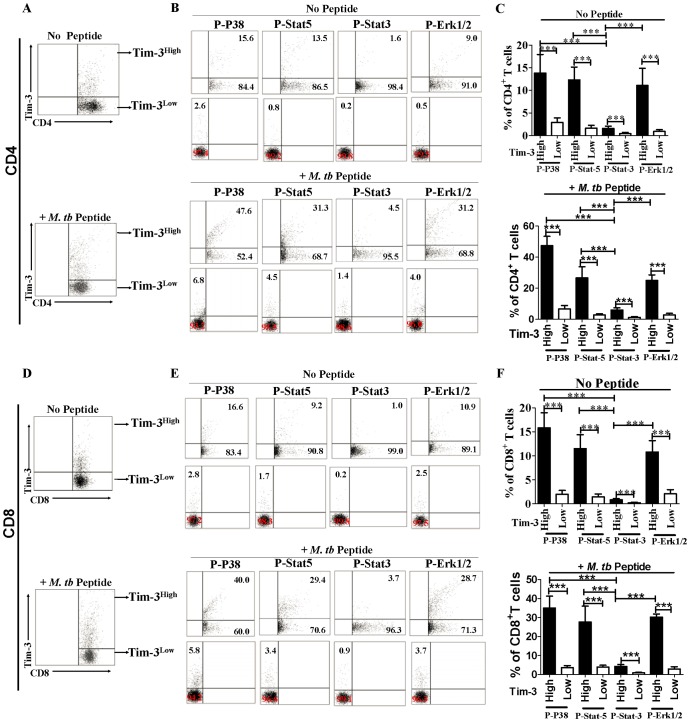
Tim-3-expressing CD4^+^ and CD8^+^ T cells from active TB patients exhibited stronger p38, stat3, stat5, and Erk1/2 signaling in cultures with or without Mtb peptide stimulation. Phosphorylation status of p38, stat5, stat3, and Erk1/2 were analyzed by polychromatic flow cytometry in Tim-3^High^ versus Tim-3^Low^ T-cell subsets. PBMC from untreated active TB patients (n = 9) were stained directly without peptide stimulation or stained after 30 min *ex vivo* stimulation with pooled Ag85-b/ESAT-6 peptides, and assessed for expression of phosphorylated(P)-p38, P-stat5, P-stat3, or P-Erk1/2 cells in Tim-3^High^ versus Tim-3^Low^ T-cell subsets using flow cytometry analysis as described above. (A) and (D) shows a representative flow cytometric gating strategy for Tim-3^High^ and Tim-3^Low^CD4^+^ and CD8+ T cells, respectively, to evaluate phosphorylated signaling molecules in cells cultured with or without *ex vivo* Mtb peptide stimulation. (B) shows representative flow cytometric dot plots demonstrating that expression levels of P-p38, P-stat5, P-stat3, and P-Erk1/2 were much higher in Tim-3^High^CD4^+^ T cells than Tim-3^Low^CD4^+^ T cells in the absence (upper panels) and presence (lower panels) of Mtb peptide stimulation. Values in dot plots indicate the percentages of expression levels of each of phosphorylated signaling molecule. Similar results were seen when Tim-3^+^ T cells were enriched by immunonmagnetic beads, and then cultured with or without Mtb peptide prior to flow analyses (data not shown). (E) is similar to (B) except for comparisons between Tim-3^High^CD8+ T cells Tim-3^Low^CD8^+^ T cells. (C) and (F) are bar graph data showing higher expression levels of P-p38, P-stat5, P-stat3, or P-Erk1/2 cells in Tim-3^High^CD4^+^ or Tim-3^High^CD8^+^ T cells than Tim-3^Low^CD4^+^ or Tim-3^Low^CD8^+^T cell subpopulations in the absence (upper panels) and presence (lower panels) of Mtb peptide stimulation (Lower panel). (C) and (F) show that percentages of expression of P-p38, P-stat5 and P-Erk1/2 are much higher than P-stat3 in Tim-3^High^CD4^+^ or Tim-3^High^CD8^+^ T cells. Data are from at least two independent experiments. *** *p<*0.001. Error bars represent SD.

Given that Tim-3^High^ CD4^+^ and CD8^+^ T-cell subsets exhibited greater effector functions of producing Th1 cytokines/cytotoxic molecules and limiting intracellular Mtb growth, we sought to determine a potential signaling mechanism underlying the Tim-3-associated enhancements. Since appropriate activation signaling is usually required for efficient T-cell effector functions in responses to Mtb infection [38], we hypothesized that greater T effector functions of Tim-3^High^ CD4^+^ and CD8^+^ T-cell subsets during Mtb infection might be driven by stronger intracellular signaling and activation. To test this hypothesis, we measured the phosphorylation of signaling molecules p38, stat3, stat5, and Erk1/2 in Tim-3^High^ CD4^+^ and CD8^+^ T-cell subsets from active TB patients in comparisons with Tim-3^Low^ control subsets. Thus, PBMC from 9 active TB patients were directly stained without *ex vivo* Mtb peptide stimulation or stimulated *ex vivo* with Mtb peptide pool, and the phosphorylated (termed P- for simplicity) p38, stat3, stat5, and Erk1/2 were immunologically stained and quantitated by flow cytometry. We found that expression levels of P-p38, P-stat3, P-stat5, and P-Erk1/2 in Tim-3^High^ CD4^+^ and CD8^+^ T-cell subsets were much higher than those in Tim-3^Low^ control subsets in cultures with or without Mtb peptide antigen stimulation (Figure 7). The finding that unstimulated Tim-3^+^, but not Tim-3^−^, T cells had higher levels of phosphorilated signal molecules was consistent with the ability of Tim-3^+^ T cells to *de novo* produce cytokines(Figure 2,3, Figure S5,S6) and to exert anti-Mtb effector function(Figure 6). Furthermore, expression levels of P-p38, P-stat5, and P-Erk1/2 were much higher than that of P-stat3 within Tim-3^High^ CD4^+^ and CD8^+^ T-cell subsets (Figure 7). Thus, Tim-3^High^ CD4^+^ and CD8^+^ T-cell subsets in active TB patients expressed higher levels of phosphorylated signaling molecules, suggesting that Tim-3-associated increases in T effector functions may involve activation signaling molecules p38, stat5, and Erk1/2 rather than stat3.

## Discussion

In the current study, we have made several interesting observations regarding Mtb infection-induced increases in Tim-3-expressing CD4^+^ and CD8^+^ T-cell subsets, Tim-3-related broad effector functions for producing Th1/Th22/cytotoxic cytokines and limiting intracellular Mtb growth, and potential mechanisms underlying Tim-3 signaling-driven enhancements of effector functions. These findings are considered novel as there are no reports, to our knowledge, for in-depth studies of Tim-3-driven T-cell immune responses in active human TB.

Interestingly, active TB patients exhibit up-regulation of Tim-3 expression and increases in Tim-3-expressing CD4^+^ and CD8^+^ T cells, and Tim-3-expressing T cells predominantly displayed a polarized effector memory phenotype (lack of expression of CCR7, CD62L, or CD27) [31]. Consistently, Tim-3-expressing Mtb-specific CD4^+^ and CD8^+^T cells in active TB patients also express high levels of another effector memory surrogate marker CD127 (IL-7 Receptor α) [39]. It is likely that effector memory phenotypes (CD127^+^ but CCR7^−^, CD62L^−^ and CD27^−^) not only favor Tim-3-expressing T cells for mounting effector functions of anti-microbial cytokine production and cytotoxicity but also facilitate these effector T cells trafficking to Mtb infection sites or inflamed lung tissues [31], [39], [40]. It is noteworthy that the predominant effector memory phenotypes of Mtb-driven Tim-3-expressing CD4^+^ and CD8^+^ T cells in active TB patients differ from virus-induced central memory phenotypic Tim-3-expressing CD8^+^ T cells in HCV- or HIV-1-infected humans [16], [19], [20]. It has been reported that Tim-3-expressing CD8^+^ T cells during HCV or HIV-1 infection display either dominant CCR7^+^ central memory or CCR7^+^ and/or CD27^+^ phenotype profiles with no or low expression of effector surrogate marker CD127 [16], [19], [20]. The discrepancy of Tim-3-expressing phenotypes between Mtb-infected and HCV-infected or HIV-1-infected patients might result from the natures of pathogens/infections and distinct immune responses to virus versus mycobacteria. A dominant central memory and a lack of preferential effector phenotypes for Tim-3-expressing T cells in HCV/HIV-1-infected patients may help to explain why the virus-driven Tim-3-expressing CD8^+^ T cells do not adequately produce effector cytokines in response to viral peptide stimulation in vitro [16], [19], [20]. In contrast, the predominant effector memory phenotypes of Tim-3-expressing CD4^+^ and CD8^+^ T cells in active TB patients are indeed consistent with enhanced effector functions for these Tim-3^High^CD4^+^ and Tim-3^High^CD8^+^ T-cell subsets.

The ability of Tim-3^+^ T cells to spontaneously produce cytokines in active TB appears to consist with our recent observation that active Mtb infection, but not SIV/SHIV infection or control setting, allows for intracellular cytokine staining (ICS) detection of cytokine production by T cells without the need for *ex vivo* stimulation with Mtb antigens [32], [33], [34]. Such an ability to *de novo* produce cytokines by T cells from Mtb-infected humans or macaques might be due to the fact that a number of T cells have differentiated into highly activated Tim-3^+^ effector cells capable of producing cytokines in response to active TB-driven immune activation and inflammation. This notion is also supported by the finding that latent Mtb infection did not induce large numbers of T cells that spontaneously produce cytokines in cultures, as only ≤2.2% and ≤3.5% cells in Tim-3^+^ T cells of LTBI subjects were able to produce cytokines after culture with medium and Mtb peptide, respectively (Figure 2, Figure 3). The high levels of Tim-3^+^/cytokine^+^ T cells in active TB might be attributed partially to the BCG vaccination background in active TB patients, as all these patients had BCG vaccination history.

The present study uncovers a surprising finding that Tim-3^High^ CD4^+^ and Tim-3^High^ CD8^+^ T-cell subsets exhibited greater effector functions for producing Th1/Th22 cytokines and CTL effector molecules. These enhanced effector functions of Tim-3-expressing T cells in TB are not totally unexpected as anti-microbial effector functions of T cells are usually linked to effector or effector memory phenotypes of these T cells [30], [31], and such enhanced effector functions appear to reflect the immunological features of effector memory phenotypes for Tim-3^High^ CD4^+^ and CD8^+^ T-cell subsets. Furthermore, enhanced effector functions of Tim-3-expressing T cells are also supported by the results from our mechanistic studies of cellular activation/signaling molecules as Tim-3^High^ CD4^+^ and CD8^+^ T-cell subsets in active TB patients expressed higher levels of phosphorylated signaling molecules p38, stat3, stat5, and Erk1/2. Despite the fact that the downstream molecular activation events for Tim-3 regulation of T cells are largely unknown [41], the up-regulated expression of phosphorylated signaling molecules in Tim-3^High^ T-cell subsets may help to explain stronger effector functions in Tim-3-expressing CD4^+^ and CD8^+^ T cells in active TB. The notion that stronger effector functions are driven by greater signaling in Tim-3-expressing T cells is also consistent with a recent finding that Tim-3 may augment T-cell signaling after a short-term stimulation [41]. This connection is also supported by the data from HIV-1-infected humans since impaired phosphorylation of above intracellular signaling molecules correlates with reduced effector functions of Tim-3-expressing CD8^+^ T cells in HIV infection [19]. A recent study reported that Tim-3-expressing CD8^+^ T cells in TB patients produced lower levels of IFN-γ than healthy controls [18]. However, subtle IFN-γ responses were detected in both TB and control groups due to the use of ESAT-6 protein, instead of peptide pool, for in vitro stimulation [18]. It is important to note that CD8^+^ T cells respond poorly to whole protein and that recombinant Mtb ESAT-6 protein actually inhibits T-cell production of IFN-γ [42]. Future studies using MHC I/peptide and MHC II/peptide tetramers will provide a better system in which to elucidate phenotypes and effector functions of Ag-specific Tim-3^+^CD4^+^ and Tim-3^+^CD8^+^ T cells.

Our results suggest that Tim-3-expressing CD4^+^ and CD8^+^ T-cell subsets possess much broader repertoire of effector functions than what was previously described. Earlier studies implicated that Tim-3 might be an exclusive cell surface marker for Th1 cells [9], and it remains unknown whether Tim-3-expressing CD4^+^ T cells in human TB could differentiate into Th22 and Th17 subsets capable of mounting immune responses to Mtb infection [32], [34]. The current study demonstrates that Tim-3-expressing CD4^+^ T cells not only can produce Th1 cytokines(IFN-γ, IL-2), but also produce appreciable amounts of IL-22, IL-4 (data not shown), and IL-17A (data not shown), suggesting that Tim-3^High^ CD4^+^ T-cell subset in active TB are capable to differentiate into Th1, Th2, Th22/Th17 cells. Interestingly, Tim-3^High^ CD8^+^ T-cell subset in active TB also exhibits broad effector functions producing the above cytokines and cytotoxic molecules. Furthermore, human Tim-3-expressing T cells can function as anti-Mtb effector cells limiting intracellular Mtb growth. Our results are consistent with a recent observation that mouse Tim-3-Gal-9 interaction can lead to inhibition of Mtb replication in macrophages [37]. The broad effector repertoires of Tim-3-expressing CD4^+^ and CD8^+^ T cells might be advantageous from the standpoints of host immune responses to Mtb infection.

The findings from our mechanistic experiments suggest that Tim-3 signaling pathways help to enhance effector functions of producing Th1, Th22 cytokines and CTL molecules. Particularly, we show that Tim-3 silencing by siRNA Tim-3 leads to reduced *de novo* production of IFN-γ and TNF-α by Tim-3-expressing T cells, and that soluble Tim-3 treatment interfering with membrane Tim-3-ligand interaction can also decrease Tim-3-driven activation and effector functions of cytokine production. On the other hand, we demonstrate that stimulation of Tim-3 signaling pathway by Ab cross-linking of membrane Tim-3 can enhance effector function of IFN-γ production by CD4^+^ and CD8^+^ T cells. These findings appear to be inconsistent with what were reported in HCV- and HIV-1-infected patients [16], [19]. It has been implicated that expression of Tim-3 on CD8^+^ T cells may be linked to progressive loss of secretion of Th1 cytokines such as IL-2, TNF-α and IFN-γ in HCV and HIV-1 infections [15], [16], [19], [21], [22]. Nevertheless, these Tim-3-associated negative effects can be explained at least partially by the phenotypic features and impaired activation signaling of Tim-3-expresssing CD8^+^ T cells in those virus-infected persons. Tim-3-expressing CD8^+^ T cells in HCV- and HIV-1-infected patients predominantly express central memory phenotypes, rather than effector and effector memory phenotypes [16], [19]; HIV-1 infection leads to impaired Stat5, Erk1/2, and p38 signaling in Tim-3-expressing CD8^+^ T cells [19]. On the contrary, active TB drives predominant effector memory phenotypes and stronger cellular activation signaling. It is likely that HCV or HIV-1 infection preferentially induces Tim-3-associated central memory or non-effector phenotypes with depression or low levels of cellular signaling, whereas Mtb infection can drive effector memory Tim-3-expressing T cells with enhanced Tim-3 signaling pathways for stronger effector functions. It is noteworthy that studies done to date have only identified Gal-9 as Tim-3 ligand [17], and precise Tim-3-induced signaling pathways remain incompletely understood [17]. From these points of views, we cannot exclude the possibility that viral and Mtb infections would engage in independent co-activation signals in T cells and induce potential different Tim-3 ligands in infected target cells.

It is currently not known whether increased numbers of Tim-3^High^ CD4^+^ and CD8^+^ T effector cells in active TB patients are detrimental or beneficial in active Mtb infection. Given that such increases are seen in the setting of active TB, Tim-3-expressing T cells might act as over-reactive effector cells and contribute to TB inflammation and pathologic lesions. This notion is supported by the finding that healthy subjects with LTBI exhibited much lower levels of Tim-3^+^CD4^+^ and Tim-3^+^CD8^+^ T effector cells producing cytokines. Over production of IFN-γ and TNF-α by Tim-3-expressing T cells may indeed elevate degree of inflammation or damages in active TB, although mouse IFN-γ and TNF-α are important for controlling Mtb infection [30], [43], [44], [45], [46], [47], [48], [49]. On the other hand, increases in Tim-3-expressing CD4^+^ and CD8^+^ T cells might result from host responses to high Mtb burden due to postprimary TB or reactivation TB. These responses, although unable to control TB, might develop as disorganized host defense or otherwise reflect protective potential if immune responses to TB can be well coordinated or if pathogenic events leading to high levels of Mtb burden can be intervened. This scenario appears to be supported by the data from the current study since Tim-3-expressing T cells can produce anti-Mtb cytokines IFN-γ/TNF-α/IL-22, and function as effector cells limiting intracellular Mtb growth in macrophages.

Thus, the current study demonstrate that Tim-3-expressing CD4^+^ and CD8^+^ T cells in active TB patients exhibit polarized effector memory phenotypes and stronger, but not impaired, anti-mycobacterium effector functions. Our findings therefore may suggest a new paradigm for T-cell immune responses regulated by Tim-3 expression in human TB, and have implications for potential immune intervention in TB.

## Materials and Methods

### Subjects

The active Mtb infection in patients was confirmed based on clinical symptoms, chest radiography, and sputum stain for acid-fast bacilli (AFB), culture and PCR for Mtb, which were done in Shenzhen Third People's Hospital. Subsequently, patients confirmed with Mtb infection received individualized regimens with rifampicine and isoniazide plus either streptomycin or ethambutol. After initiation of TB treatment, patients were evaluated again clinically and bacteriologically to determine the effectiveness of the therapy and the transition of disease. Healthy controls (HCs) are a cohort of individuals negative for tuberculin skin test (TST) with no bacteriological and clinical evidence of TB disease. Subjects with LTBI are a cohort of individuals strongly positive for TST with no bacteriological and clinical evidence of active TB disease. All samples of Mtb-infected individuals or healthy controls were collected with informed written consent according to protocols approved by the Internal Review and the Ethics Boards of Shenzhen Third People's Hospital and Zhongshan School of Medicine of Sun Yat-sen University.

### Monoclonal antibodies (mAbs), protein and peptides

Abs against the following molecules were used: CD3-FITC, CD3-PE, CD3-APC, or CD3-PE/Cy7 (Clone OKT3, ebioscience); CD4-APC, CD4-PE, CD4-PE/Cy7, or CD4-Biotin (Clone RPA-T4, BD); CD8-APC, CD8-PE, CD8-PE/Cy7, or CD8-Biotin (Clone RPA-T8, BD); Tim-3-PE, Tim-3-Alexa fluor488 (Clone 344823, R&D); CCR7(CD197)-FITC (Clone 3D12, ebioscience), CD27-APC (Clone O323, ebioscience), CD45RA-PE/Cy7 (Clone HI100, ebioscience), CD127-FITC (Clone eBioRDR5, ebioscience), CD62L-PE (Clone DREG56, ebioscience); TNF-α-APC, TNF-α-FITC(Clone Mab11, ebioscience); IFN-γ-FITC, IFN-γ-APC (Clone 4s.b3, ebioscience); Granzyme B-FTIC (Clone GB11, BD), perforin-FITC (Clone deltaG9, ebioscience); IL-2-FITC (Clone MQ1-17, BD), IL-4-APC (Clone 8D4-8, ebioscience), IL-17a(Clone eBio64DEC17, ebioscience), IL-22-PE (Clone 22URTI, ebioscience), PE-anti-Galectin-9(Clone 9M1-3,biolegend), anti-IL-1β (Clone AS10, BD). Streptavidin-Phycoerythrin-Texas Red (BD) was used to conjugate CD4-biotin or CD8-biotin antibody. Recombinant human Tim-3 Fc Chimera (i.e.Tim-3-Ig) and the purified Ab for Tim-3 (Clone 344823) were both from R&D. Overlapping Mtb Ag85-b/ESAT-6 pooled peptides(15 a.a. overlapped by 12 spanning entire Ag85 or ESAT6 protein were synthesized and used as we previously described [34]. The purpose choosing combined peptides for ICS was to maximize detection of Mtb-specific T effector cells and to optimally work with a limited amount of blood volume collected from individual subjects.

### Isolation of peripheral blood mononuclear cells (PBMC), intracellular cytokine staining (ICS) and flow cytometric analysis

PBMC were isolated from whole blood by Ficoll (GE health) density gradient centrifugation, as we described previously [32]. Intracellular cytokine staining (ICS) was done as we previously described [32]. We used two approaches for ICS: (i) PBMC from untreated active TB patients were stimulated *ex vivo* with Mtb peptides pool, and then stained for Tim-3 and anti-Mtb effector cytokines including IFN-γ, TNF-α, IL-2, and IL-22 and analyzed by polychromatic flow cytometry.. (ii) PBMC from the same TB patients were directly stained for the above cytokines without peptide stimulation as we recently described [32], [34]. The specificity and utility of the direct intracellular cytokine staining approach has been validated during Mtb infection of macaques and humans as well as in the control settings [32], [33], [34]. Briefly, PBMC were cultured with or without re-stimulation with Mtb Ag85-b/ESAT-6 peptide pools for 6 hours in presence of brefeldin A (5 µg/ml; BD) in the final 3 hours of culture. Cells were then fixed, permeabilized and washed with the Perm/Wash buffer (BD). For PBMC without *in vitro* antigenic re-stimulation, cells were fixed, permeabilized and washed with the BD Perm/Wash buffer. After permeabilization, cells were stained using fluochrome-conjugated mAbs or isotype control Abs. Data were acquired on Beckman Coulter Cytomics FC500 (Beckman) and analyzed with CXP (Beckman) software.

### Ligand competition assay using soluble form of Tim-3(s-Tim-3)

PBMC derived from TB patients were incubated with 2 µg/ml soluble form of Tim-3 molecules (human Tim-3 Fc Chimera, purchased from R&D) in presence or absence of Mtb Ag85-b/ESAT-6 pooled peptides for 6 days. Production of IFN-γ and TNF-α by Tim-3-expressing T cells were then analyzed using ICS protocol and flow cytomery.

### Tim-3 stimulation assay using cross-linking Tim-3-specific monoclonal Ab (mAb)

PBMC from subjects with active TB patients were incubated with 10 µg/ml of purified mouse anti-human mAb against Tim-3 (purchased from R&D) or isotype control IgG (10 µg/ml) in presence or absence of Mtb Ag85-b/ESAT-6 pooled peptides for 6 days. The effects of Tim-3 mAb stimulation on the production of IFN-γ and TNF-α by Tim-3-expressing T cells were then analyzed using ICS protocol and flow cytometry.

### Staining of intracellular signaling molecules

PBMC from active TB patients were stimulated with Mtb Ag85-b/ESAT-6 pooled peptides for 30 mins. After fixation and subsequent washing, cells were permeabilized with Perm/Wash buffer (BD). Cells were washed and stained with isotype control Ab or fluochrome-conjugated phosphospecific Abs: P-stat3 (pY705)-PE (Clone 4/P-STAT3, BD), P-erk1/2(pT202/pY204)-Alexa Fluor488 (Clone 20A, BD), P-p38 (pT180/pY182)-Alexa fluor 647 (Clone 36, BD), or P-stat5 (pY694)-FITC (Clone 47, BD).

### Knockdown of Tim-3 via siRNA

PBMC derived from TB patients were transiently transfected with 20 nM siRNA targeting Tim-3 (si-Tim-3) or 20 nM nontargeting siRNA (si-control) using Lipofectamine 2000 (Invitrogen). siRNA targeting Tim-3 (si-Tim-3) and nontargeting siRNA (si-control) are commercially available from Ribobio (Guangzhou, China). Knockdown efficiency was analyzed by real-time PCR or flow cytometry 48 hours after transfection. At 2 days after transfection, PBMC were cultured in presence or absence of Mtb Ag85-b/ESAT-6 peptide pools for 6 days, and cytokine production was analyzed by ICS and flow cytometry.

### Isolation of monocytes and Tim-3^+^CD3^+^ T cells

Cell isolation was done as we described previously [34]. Briefly, PBMC were isolated from the blood of active TB patients, and monocytes were obtained by adherence purification on plastic plates. The plates were washed after 2 hours of adherence, and monocytes were detached by cold 2% FBS/PBS. The non-adherent cell fraction containing T cells was stained with anti-Tim-3-PE (ebioscience), followed by anti-PE magnetic beads (Miltenyi Biotec). The stained live cells were then loaded to the purification column following instructions from the manufacturer. The passing fraction was collected as Tim-3**^−^** (negative) cells that did not bear Tim-3; Tim-3**^+^** T cells held by anti-PE magnetic beads were then released by releasing buffer (Miltenyi Biotec). The isolated Tim-3**^+^** T cells were stained again with anti-CD3 FITC (BD), followed by anti-FITC magnetic microbeads for secondary purification. The purity of isolated Tim-3**^+^**CD3**^+^** T cells and Tim-3**^−^**CD3**^+^** T cells is over 95% ([34], data not shown).

### *In vitro* Mtb infection of monocytes-derived macrophages and intracellular Mtb growth assay

This was done similarly like what we recently described [34]. Briefly, autologous monocytes (5×10^4^/well) were cultured in round-bottom 96-well plates with 10% FBS-RPMI 1640 medium in presence of human rIL-4 (BD) and GM-CSF (Sigma-Aldrich) for 8 days. Supernatants were then removed, and Mtb (H37Ra) inoculum was added at a MOI = 1. After overnight infection at 37°C, supernatants were aspirated and each well was washed extensively to remove extracellular Mtb. Enriched Tim-3^+^CD3^+^ T cells (5×10^5^/well) or Tim-3^−^CD3^+^ T cells (5×10^5^/well) were incubated with Mtb-infected macrophages (MΦs) in presence or absence of anti-IL-1β Ab (10 µg/ml) or IgG (10 µg/ml). After culturing in 5% CO_2_ at 37°C for 4 days, wells were aspirated, and lysis buffer (0.067% SDS in Middlebrook 7H9) was added to each well. Plates were incubated at 37°C, followed by neutralization of SDS with PBS with 20% BSA. Lysates from each well were pooled, and two 10-fold serial dilutions of lysate in 7H9 medium were made. Aliquots of each dilution of lysate and supernatant were plated onto Middlebrook 7H10 agar and incubated until colonies were large enough to be counted.

### Statistical analysis

Statistical significance was determined with Student *t-test* (difference between two groups or conditions), and a *p* value <0.05 in all cases was considered statistically significant (95% confidence interval), as we described previously [32]. Analysis was performed using Prism 5.0 software (GraphPad Software, Inc.).

## Supporting Information

Figure S1 **TB infection led to up-regulation of Galectin-9 (Gal-9) expression and increases in numbers of Gal-9-expressing CD4^+^ and CD8^+^ T cells.** PBMCs were stained using ICS protocol. (A) is representative flow cytometric dot plots showing Gal-9 expression in a healthy control (HC), a representative individual with LTBI, or a typical individual with untreated active TB disease. No Gal-9 expression was observed when we used isotype matched IgG to stain PBMCs (Data not shown). Values in the upper right quadrant indicate the percentages of Gal-9-expressing CD4^+^ and CD8^+^ T cells. Data were gated on CD3^+^CD4^+^ and CD3^+^CD8^+^, respectively. (B) and (C) are pooled flow cytometric data showing that the percentages (%) of Gal-9 expression on CD4^+^ and CD8^+^ T cells from 9 subjects with active TB disease are much higher than either 9 subjects with LTBI or 9 healthy control (HCs). Horizontal bars depict the mean percentage of Mtb-specific Gal-9 expression on CD4^+^ and CD8^+^ T cells. *** *p*<0.001, ** *p*<0.01, *p<0.05.
(PDF)

Figure S2 **Tim-3-expressing CD4^+^ and CD8^+^ T cells in active TB patients preferentially exhibited effector memory, but not central memory, phenotypes.** PBMCs isolated from untreated active TB patients (n = 9) were cultured with or without *ex vivo* stimulation of pooled Ag85-b/ESAT-6 peptides, stained with fluorochrome-conjugated mAbs, analyzed by polychromatic flow cytometry. (A) is representative flow cytometric dot plots showing the CCR7 and CD45RA expression in Tim-3-expressing CD4^+^ and CD8^+^ T cells from a Mtb-infected individual with untreated active TB disease (gated on CD3^+^CD4^+^Tim-3^+^ and CD3^+^CD8^+^Tim-3^+^, respectively). Values in each quadrant indicate the percentages of CD45RA^+^CCR7^+^, CD45RA^−^CCR7^+^, CD45RA^−^CCR7^−^, CD45RA^+^CCR7^−^ cells. (B) and (C) are pooled data showing the preferential expression of CD45RA^−^CCR7^−^ effector memory phenotype in Tim-3-expressing CD4^+^ and CD8^+^ T cells of Mtb-infected individuals (n = 9). Data shown are representative of at least three independent experiments. * *p*<0.05, ** *p*<0.01, *** *p*<0.001.
(PDF)

Figure S3 **M.tb-specific CD4^+^ and CD8^+^ T cells expressing Tim-3 lack expression of CD27 and CD62L, but have higher expression levels of CD127.** PBMCs of Mtb-infected individuals with untreated active TB disease (n = 9) were stained directly or re-stimulated using pooled Ag85-b/ESAT-6 peptides. (A), (D), and (G) are representative flow cytometric dot plots (gated on CD3+CD4+) showing that less CD27 (A) and CD62L molecule (D), but more CD127 (G) expressed on Tim-3-expressing Mtb-specific CD4^+^ and CD8^+^ T cells. Numbers in upper right quadrant of each flow cytometric dot plot indicate the percentages of CD27^+^Tim-3^+^,CD62L^+^Tim-3^+^, or CD127^+^Tim-3^+^ cells. (B), (C), (E), (F), (H), and (I) are pooled data showing that Tim-3-expressing Mtb-specific CD4^+^ and CD8^+^ T cells contained much smaller percentages of CD27^+^ or CD62L^+^ T cells, but greater percentages of CD127^+^ T cells. Data shown are representative of at least three independent experiments. Error bars represent SD. *** *p*<0.001.
(PDF)

Figure S4 **Tim-3-expressing CD4^+^ and CD8^+^ T cells in subjects with active TB disease or LTBI preferentially exhibited CD27^−^CD45RA^−^ phenotypes.** PBMCs isolated from subjects with untreated active TB disease (n = 9) or with LTBI (n = 9) were cultured with or without *ex vivo* stimulation of pooled Ag85-b/ESAT-6 peptides, stained with fluorochrome-conjugated mAbs, analyzed by polychromatic flow cytometry. (A) is representative flow cytometric dot plots showing the expression of CD27 and CD45RA in Tim-3-expressing CD4^+^ and CD8^+^ T cells from a Mtb-infected individual with untreated active TB disease (gated on CD3^+^CD4^+^Tim-3^+^ and CD3^+^CD8^+^Tim-3^+^, respectively). (D) Similar representative flow cytometric dot plot show the expression of CD27 and CD45RA in a subject with LTBI. Values in each quadrant indicate the percentages of CD45RA^+^CD27^+^, CD45RA^−^CD27^+^, CD45RA^−^CD27^−^, CD45RA^+^CD27^−^ cells. (B) and (C) Pooled data show the preferential expression of CD27^−^CD45RA^−^ phenotype in Tim-3-expressing CD4^+^ and CD8^+^ T cells derived from the individuals with active TB (n = 9). Data shown are representative of at least three independent experiments. (E) and (F) show the similar pooled data in LTBI. Error bars represent SD. *** *p*<0.001.
(PDF)

Figure S5 **Tim-3^High^ CD4^+^ T cells show much stronger Mtb-specific TNF-α, IL-2, and IL-22 responses than their Tim-3^Low^ counterparts.** PBMCs derived from 9 TB patients with untreated active TB disease or 9 individuals with LTBI were stimulated, stained, and analyzed as like CD4^+^ T cells shown in Figure 2. Tim-3^High^ and Tim-3^Low^ populations were gated to analyze the relationship between Tim-3 expression and IL-2, IL-22 and TNF-α responses of CD4^+^ T cells. Numbers in each of dot plots represent the percentages of IL-2-, IL-22-, or TNF-α-producing CD4^+^ T cells. (A), (C), and (E) are representative flow cytometric dot plots showing *de novo* and Mtb-specific cytokine responses of TNF-α, IL-2 ,and IL-22 in Tim-3^High^CD4^+^ T cell and Tim-3^Low^CD4^+^ T cells from an individual with active TB disease. (G), (I), and (K) shows that similar responses of TNF-α, IL-2, and IL-22 were also observed in Tim-3^High^CD4^+^ T cell and Tim-3^Low^CD4^+^ T cells from an individual with LTBI. (B) is pooled flow cytometric data from individuals with active TB disease (n = 9) show that the percentages of TNF-α^+^CD4^+^T cells are much higher in Tim-3^High^CD4^+^T cells than those in Tim-3^Low^CD4^+^T cells. Similar pooled flow cytometric data in (D) and (F), respectively, show that Tim-3^High^CD4^+^T cells contained much higher percentages of IL-2^+^CD4^+^T cells or IL-22^+^CD4^+^T cells than their Tim-3^Low^ counterparts. (H), (J), and (L) are similar pooled data from 9 individuals with LTBI show that Tim-3^High^CD4^+^T cells contained much higher percentages of TNF-α^+^CD4^+^T cells, IL-2^+^CD4^+^T cells , and IL-22^+^CD4^+^T cells than their Tim-3^Low^ counterparts. Data shown are representative of at least three independent experiments. Error bars represent SD. *** *p*<0.001, * *p*<0.05.
(PDF)

Figure S6 **Tim-3^High^ CD8^+^ T cells show stronger** ***de novo*** **or Mtb-specific responses of TNF-α and IL-2 than Tim-3^Low^CD8^+^ T cells.** PBMCs derived from 9 individuals with untreated active TB disease or 9 individuals with LTBI were stimulated, stained, and analyzed using the same protocol as shown in Figure 2. Tim-3^High^ and Tim-3^Low^ populations were gated to analyze the relationship between Tim-3 expression and TNF-α and IL-2 responses of CD8^+^ T cells. Numbers in dot plots indicate the percentages of TNF-α- or IL-2-expressing CD8^+^ T cells. (A) and (C) are representative flow cytometric dot plots show *de novo* or Mtb-specific TNF-α and IL-2 responses in Tim-3^High^CD8^+^ T cell and Tim-3^Low^CD8^+^ T cells from an individual with active TB disease. (E) and (G) are similar representative flow cytometric dot plots showing the similar *de novo* or Mtb-specific TNF-α and IL-2 responses in Tim-3^High^CD8^+^ T cell and Tim-3^Low^CD8^+^ T cells from an individual with LTBI. (B) and (D) are pooled flow cytometric data from individuals with active TB disease showing that the percentages of either TNF-α^+^CD8^+^T cells or IL-2^+^CD8^+^ T cells are much higher in Tim-3^High^CD8^+^T cells, as compared to Tim-3^Low^CD8^+^T cells (n = 9). (F) and (H) are similar pooled data from individuals with LTBI show that the percentages of either TNF-α^+^CD8^+^T cells or IL-2^+^CD8^+^ T cells are much higher in Tim-3^High^CD8^+^T cells, as compared to Tim-3^Low^CD8^+^T cells (n = 9). Data shown are representative of at least three independent experiments. Error bars represent SD.* *p*<0.05, ** *p*<0.01, *** *p*<0.001.
(PDF)

Figure S7 **Tim-3 expression is associated with stronger granzyme B response of Mtb-specific CD4^+^ and CD8^+^ T cells.** PBMCs derived from individuals with untreated active TB disease (n = 9) or with LTBI (n = 9) were cultured in presence or absence of pooled Ag85-b/ESAT-6 peptides, and analyzed as like perforin. (A) is representative flow cytometric dot plots showing the percentages of Mtb-specific or *de novo* production of granzyme with or without *ex vivo* stimulation of pooled Ag85-b/ESAT-6 peptides in CD4^+^ (or CD8^+^) T cells from an individual with untreated active TB disease. A two-tiered gating system was used as well to analyze Mtb-specific or *de novo* perforin production by Tim-3-expressing CD4^+^ (or CD8^+^) T cells, considering Tim-3^High^ and Tim-3^Low^ subpopulations. Numbers in each of dot plot represent the percentages of granzyme B-producing CD4^+^ or CD8^+^ T cells. (D) is similar flow cytometric dot plots that were shown to analyze granzyme B expression in CD4^+^ (or CD8^+^) T cells from an individual with LTBI. (B) and (C) are pooled flow cytometric data from individuals with active TB disease (n = 9) showing that the percentages of granzyme B^+^CD4^+^ (or CD8^+^) T cells are much higher in Tim-3^High^CD4^+^ (or CD8^+^) T cells than their Tim-3^Low^ counterparts. (E) and (F) are similar pooled flow cyotmetry data from individuals with LTBI (n = 9) showing that the percentages of granzyme B^+^CD4^+^ (or CD8^+^) T cells are much higher in Tim-3^High^CD4^+^ (or CD8^+^) T cells than their Tim-3^Low^ counterparts. Data shown are representative of at least three independent experiments. Error bars represent SD. * *p*<0.05, ** *p*<0.01, *** *p*<0.001.
(PDF)

Figure S8 **Tim-3 expression is associated with stronger degranulation capability of Mtb-specific CD4^+^ and CD8^+^ T cells.** PBMCs derived from Mtb-infected individuals (n = 9) with untreated active TB disease were cultured in presence or absence of pooled Ag85-b/ESAT-6 peptides, and stained with fluorochrome-conjugated mAbs, followed by analysis using polychromatic flow cytometry. (A) is representative flow cytometric plots showing the percentages of CD107a expression with or without *ex vivo* stimulation of pooled Ag85-b/ESAT-6 peptides in Mtb-specific CD4^+^ and CD8^+^ T cells from an Mtb-infected individual with untreated active TB disease. (B) is complied flow cytometric data from Mtb-infected individuals (n = 9) showing that the percentages of Tim-3^High^CD4^+^ T cells are much higher in CD107a^+^ T cells than those in CD107a^−^ T cells. (C) and (D) are representative flow cytometric data and summary bar graphic data, respectively, showing that similar stronger CD107a expression as like Tim-3^High^CD4^+^ T cells were also observed for Tim-3^High^CD8^+^ T cells. Data shown are representative of at least three independent experiments. Error bars represent SD. * *p*<0.05, **p<0.01, *** *p*<0.001.
(PDF)

Figure S9 **Stimulation of Tim-3 pathway using anti-Tim-3 Ab enhances the Mtb-specific effector functions of CD4^+^ and CD8^+^ T cells.** PBMCs derived from Mtb-infected individuals (n = 9) with untreated active TB disease were stimulated *ex vivo* using anti-Tim-3 mAb (10 µg/ml) or isotype control Ab (10 µg/ml) in presence or absence of pooled Ag85-b/ESAT-6 peptides for 6 days. Cells were then stained using ICS protocol, and analyzed by flow cytometry. (A) is typical flow cytometric dot plots showing the effect of Tim-3 stimulation using anti-Tim-3 mAb on Mtb-specific IFN-γ response of Tim-3^High^CD4^+^ T cells. Numbers in dot plots in each of sub-figure show the percentages of IFN-γ^+^Tim-3^High^CD4^+^ T cells. (C) is summary bar graphic data showing that Tim-3 stimulation using anti-Tim-3 mAb but not isotype control Ab significantly enhances the production of Mtb-specific IFN-γ by Tim-3^High^CD4^+^ T cells. (B) and (D) show that similar enhancement of Mtb-specific IFN-γ responses upon stimulation with anti-Tim-3 mAb (10 µg/ml) were also observed in Tim-3^High^CD8^+^ T cells. Data shown are representative of at least three independent experiments. Error bars represent SD. ** *p*<0.01, *** *p*<0.001, NS, no statistical significance.
(PDF)

Table S1 **Clinical characteristics of the enrolled subjects.**
(PDF)
