# Supplementary figures and images for: Tim-3-Expressing CD4+ and CD8+ T Cells in Human Tuberculosis (TB) Exhibit Polarized Effector Memory Phenotypes and Stronger Anti-TB Effector Functions
Source: PLoS Pathog. 2012 Nov 8;8(11):e1002984. doi: 10.1371/journal.ppat.1002984 (PMC3493466; doi:10.1371/journal.ppat.1002984)

# Figure S1

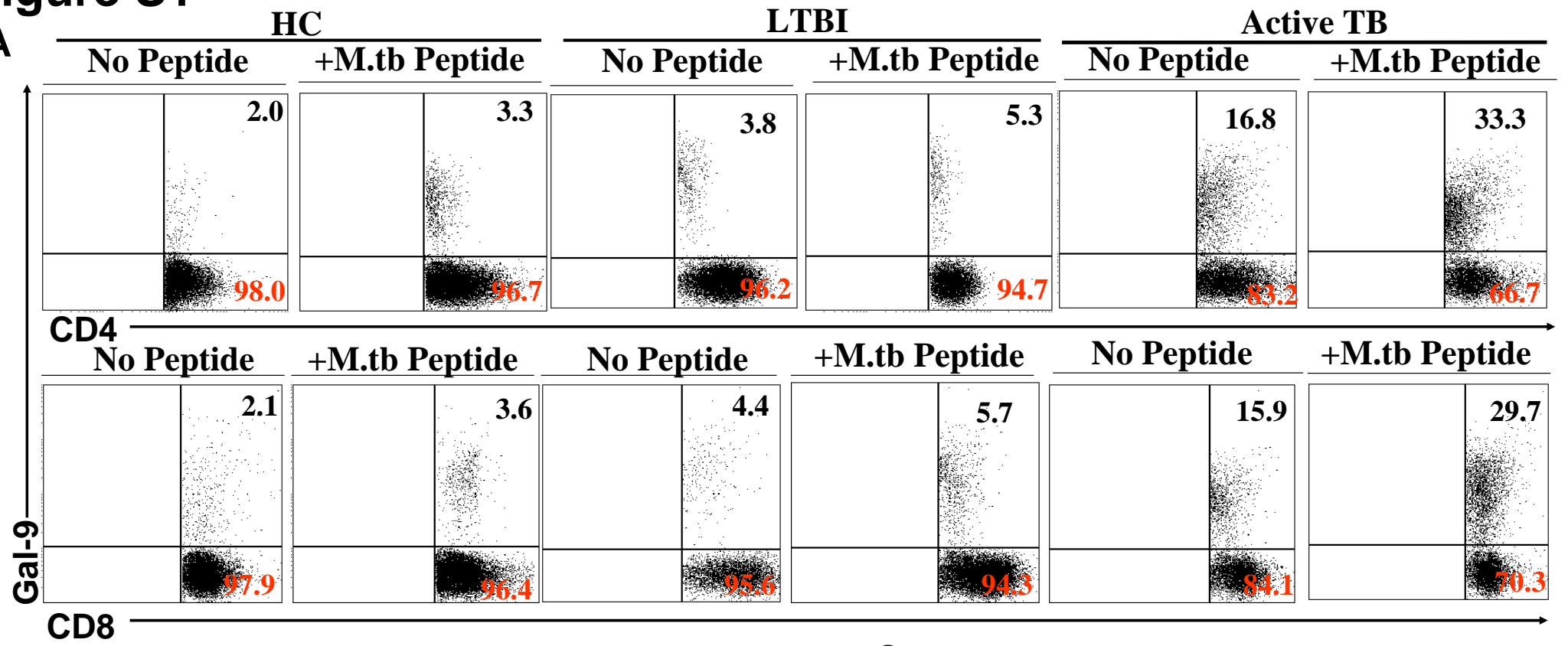

**B**

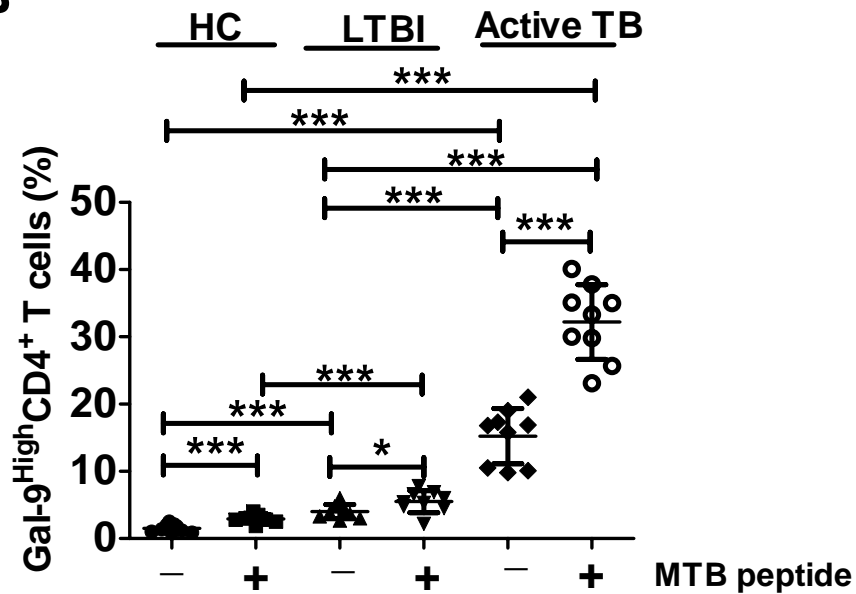

**C**

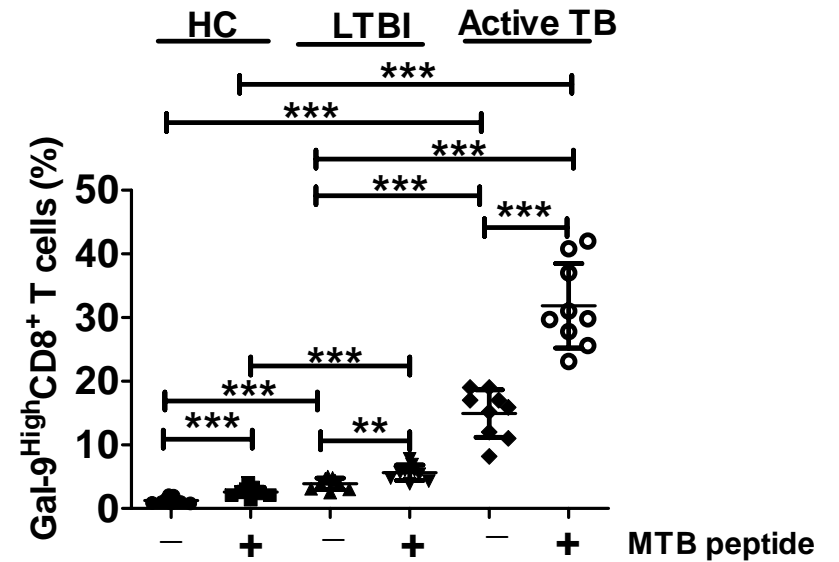

Supplement: Figure S1 — TB infection led to up-regulation of Galectin-9 (Gal-9) expression and increases in numbers of Gal-9-expressing CD4+ and CD8+ T cells. PBMCs were stained using ICS protocol. (A) is representative flow cytometric dot plots showing Gal-9 expression in a healthy control (HC), a representative individual with LTBI, or a typical individual with untreated active TB disease. No Gal-9 expression was observed when we used isotype matched IgG to stain PBMCs (Data not shown). Values in the upper right quadrant indicate the percentages of Gal-9-expressing CD4+ and CD8+ T cells. Data were gated on CD3+CD4+ and CD3+CD8+, respectively. (B) and (C) are pooled flow cytometric data showing that the percentages (%) of Gal-9 expression on CD4+ and CD8+ T cells from 9 subjects with active TB disease are much higher than either 9 subjects with LTBI or 9 healthy control (HCs). Horizontal bars depict the mean percentage of Mtb-specific Gal-9 expression on CD4+ and CD8+ T cells. *** p<0.001, ** p<0.01, *p<0.05. (PDF) [file ppat.1002984.s001.pdf]

**Figure S2**

**A**

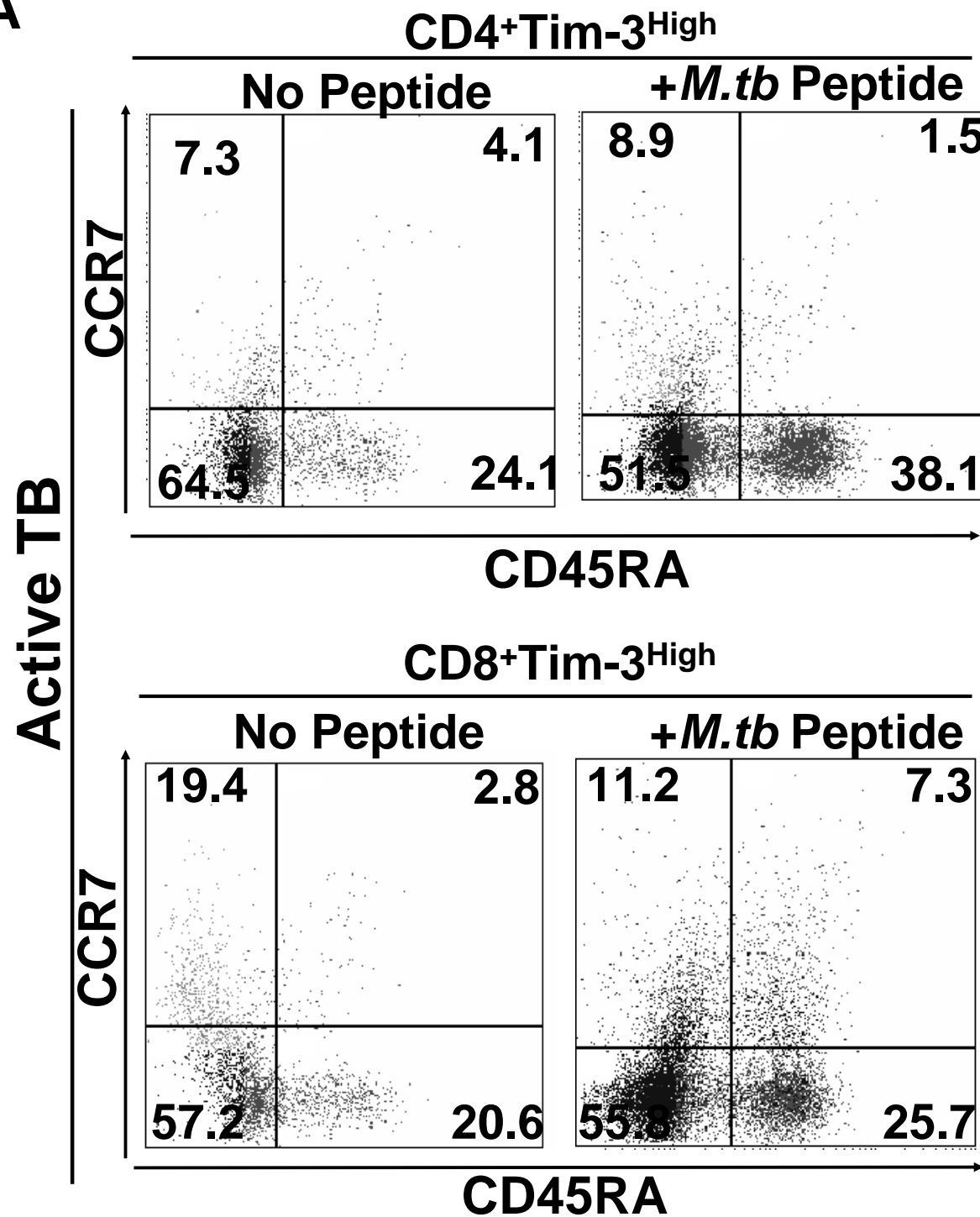

**B**

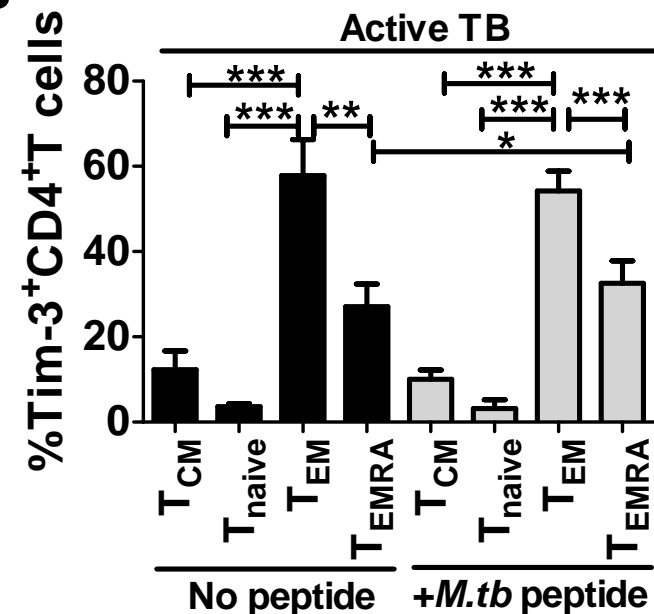

**C**

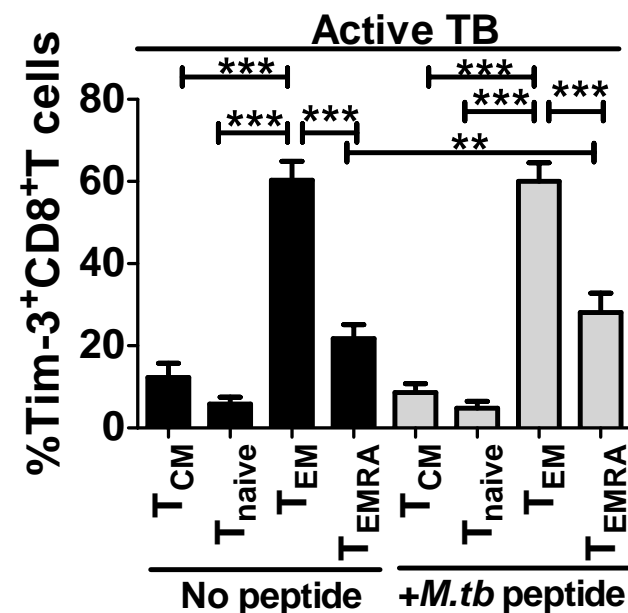

Supplement: Figure S2 — Tim-3-expressing CD4+ and CD8+ T cells in active TB patients preferentially exhibited effector memory, but not central memory, phenotypes. PBMCs isolated from untreated active TB patients (n = 9) were cultured with or without ex vivo stimulation of pooled Ag85-b/ESAT-6 peptides, stained with fluorochrome-conjugated mAbs, analyzed by polychromatic flow cytometry. (A) is representative flow cytometric dot plots showing the CCR7 and CD45RA expression in Tim-3-expressing CD4+ and CD8+ T cells from a Mtb-infected individual with untreated active TB disease (gated on CD3+CD4+Tim-3+ and CD3+CD8+Tim-3+, respectively). Values in each quadrant indicate the percentages of CD45RA+CCR7+, CD45RA−CCR7+, CD45RA−CCR7−, CD45RA+CCR7− cells. (B) and (C) are pooled data showing the preferential expression of CD45RA−CCR7− effector memory phenotype in Tim-3-expressing CD4+ and CD8+ T cells of Mtb-infected individuals (n = 9). Data shown are representative of at least three independent experiments. * p<0.05, ** p<0.01, *** p<0.001. (PDF) [file ppat.1002984.s002.pdf]

Figure S3

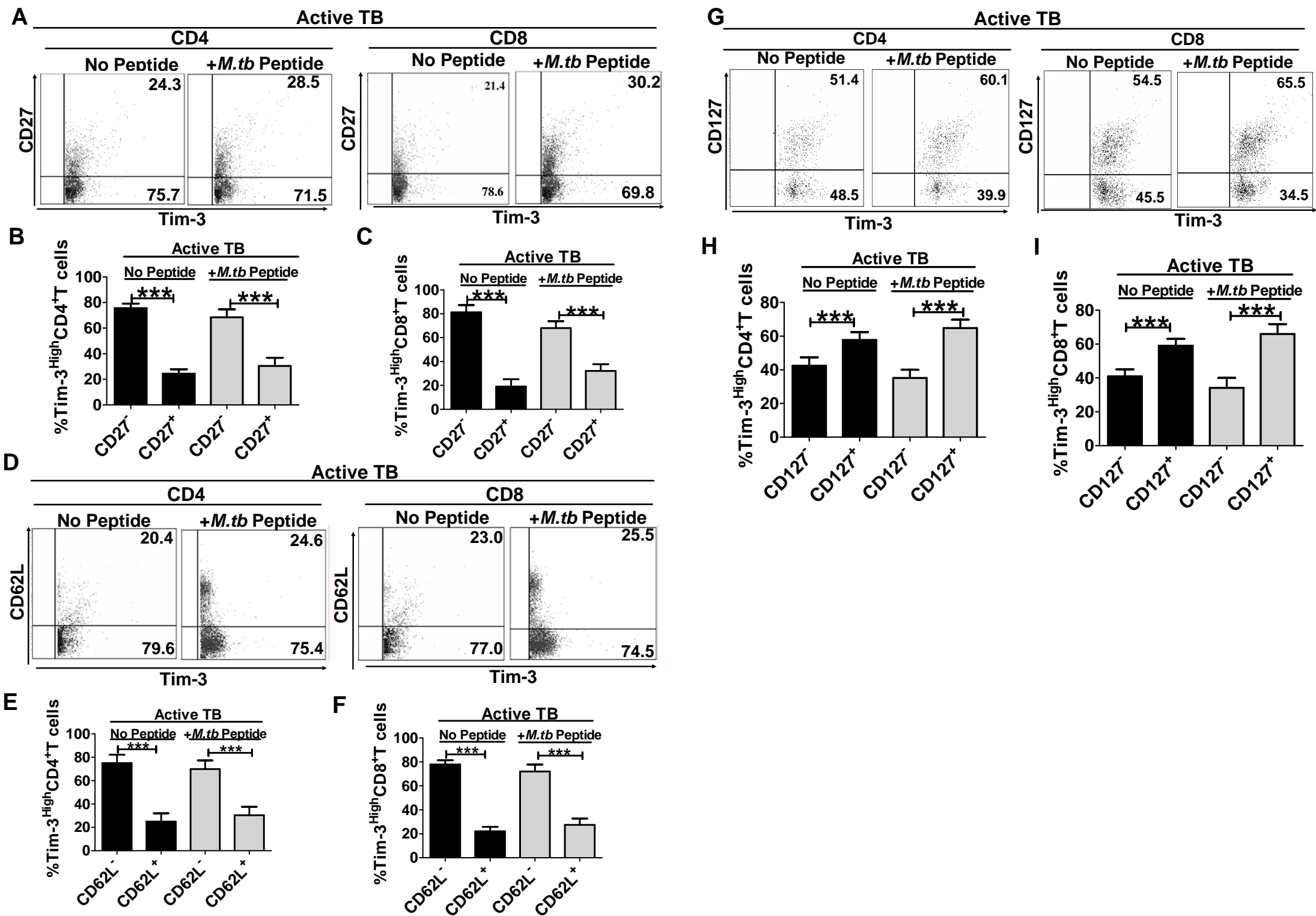

Supplement: Figure S3 — M.tb-specific CD4+ and CD8+ T cells expressing Tim-3 lack expression of CD27 and CD62L, but have higher expression levels of CD127. PBMCs of Mtb-infected individuals with untreated active TB disease (n = 9) were stained directly or re-stimulated using pooled Ag85-b/ESAT-6 peptides. (A), (D), and (G) are representative flow cytometric dot plots (gated on CD3+CD4+) showing that less CD27 (A) and CD62L molecule (D), but more CD127 (G) expressed on Tim-3-expressing Mtb-specific CD4+ and CD8+ T cells. Numbers in upper right quadrant of each flow cytometric dot plot indicate the percentages of CD27+Tim-3+,CD62L+Tim-3+, or CD127+Tim-3+ cells. (B), (C), (E), (F), (H), and (I) are pooled data showing that Tim-3-expressing Mtb-specific CD4+ and CD8+ T cells contained much smaller percentages of CD27+ or CD62L+ T cells, but greater percentages of CD127+ T cells. Data shown are representative of at least three independent experiments. Error bars represent SD. *** p<0.001. (PDF) [file ppat.1002984.s003.pdf]

Figure S4

A

Active TB

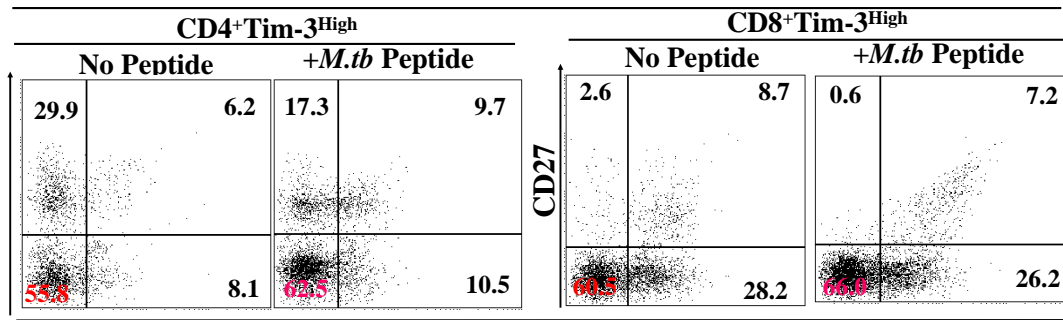

B

CD45RA

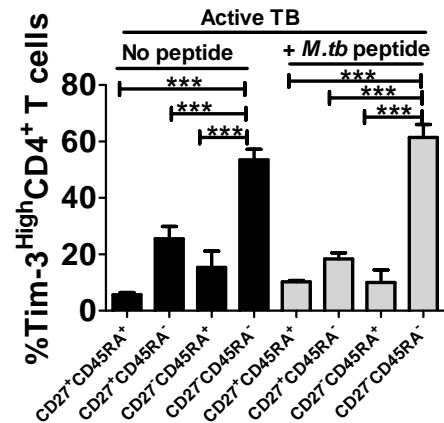

C

Active TB

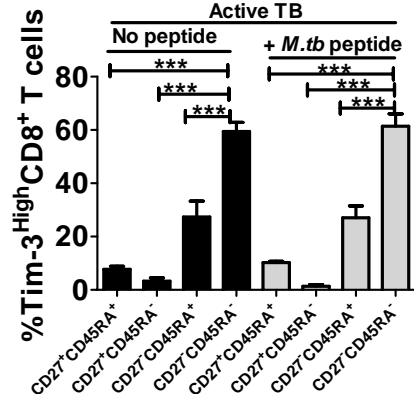

D

LTBI

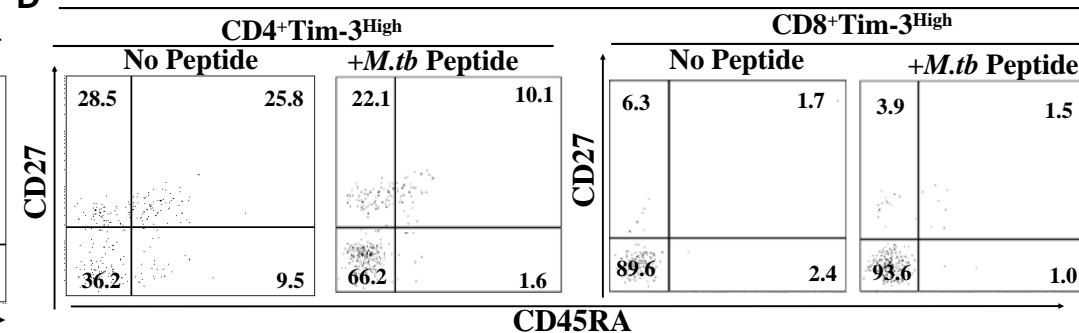

E

LTBI

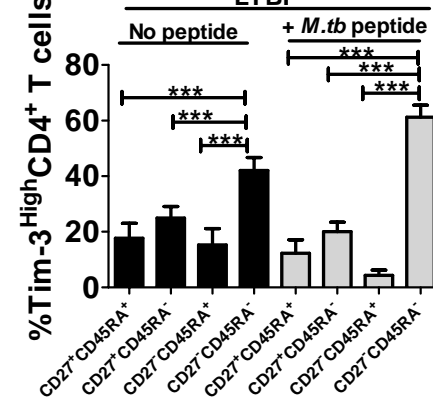

F

LTBI

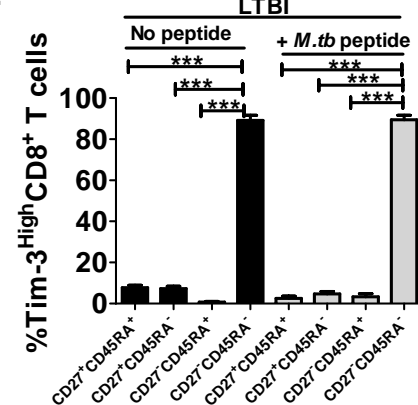

Supplement: Figure S4 — Tim-3-expressing CD4+ and CD8+ T cells in subjects with active TB disease or LTBI preferentially exhibited CD27−CD45RA− phenotypes. PBMCs isolated from subjects with untreated active TB disease (n = 9) or with LTBI (n = 9) were cultured with or without ex vivo stimulation of pooled Ag85-b/ESAT-6 peptides, stained with fluorochrome-conjugated mAbs, analyzed by polychromatic flow cytometry. (A) is representative flow cytometric dot plots showing the expression of CD27 and CD45RA in Tim-3-expressing CD4+ and CD8+ T cells from a Mtb-infected individual with untreated active TB disease (gated on CD3+CD4+Tim-3+ and CD3+CD8+Tim-3+, respectively). (D) Similar representative flow cytometric dot plot show the expression of CD27 and CD45RA in a subject with LTBI. Values in each quadrant indicate the percentages of CD45RA+CD27+, CD45RA−CD27+, CD45RA−CD27−, CD45RA+CD27− cells. (B) and (C) Pooled data show the preferential expression of CD27−CD45RA− phenotype in Tim-3-expressing CD4+ and CD8+ T cells derived from the individuals with active TB (n = 9). Data shown are representative of at least three independent experiments. (E) and (F) show the similar pooled data in LTBI. Error bars represent SD. *** p<0.001. (PDF) [file ppat.1002984.s004.pdf]

Figure S5

Active TB

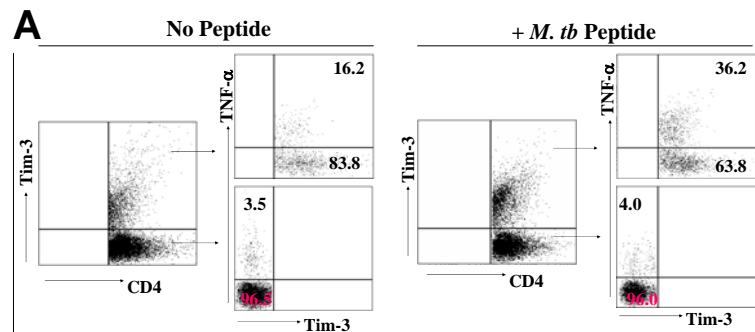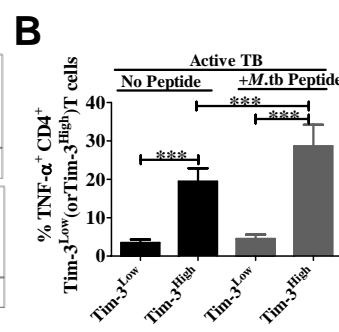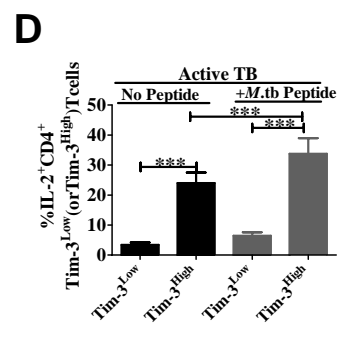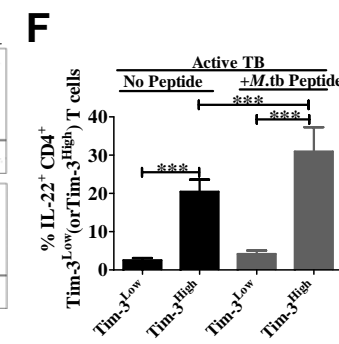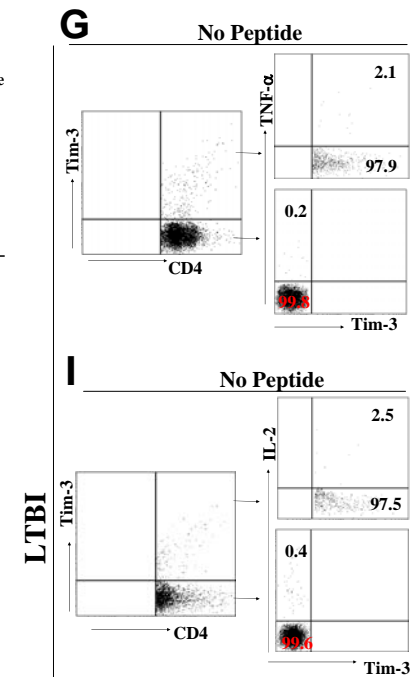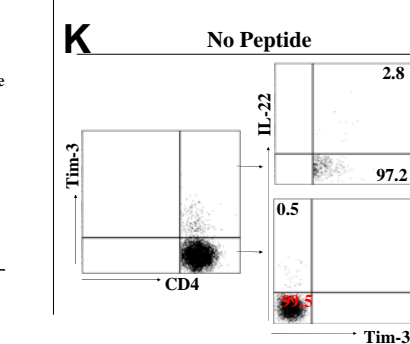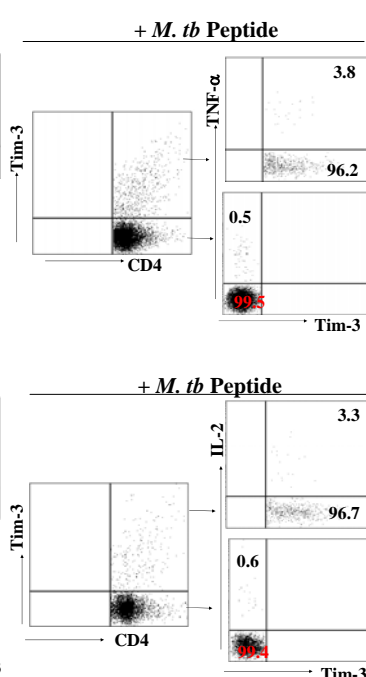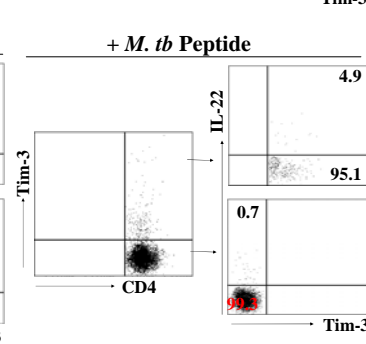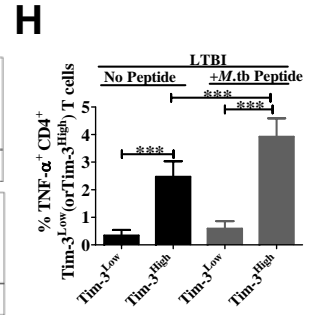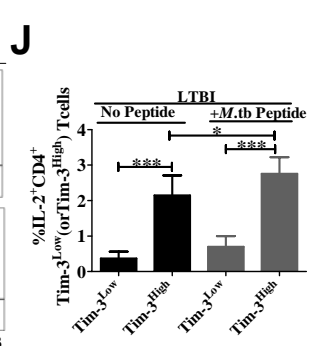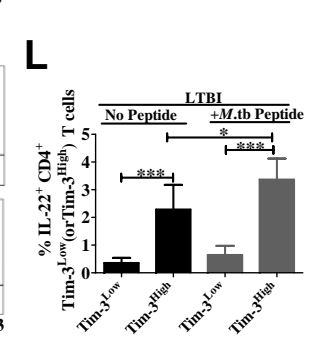

Supplement: Figure S5 — Tim-3High CD4+ T cells show much stronger Mtb-specific TNF-α, IL-2, and IL-22 responses than their Tim-3Low counterparts. PBMCs derived from 9 TB patients with untreated active TB disease or 9 individuals with LTBI were stimulated, stained, and analyzed as like CD4+ T cells shown in Figure 2. Tim-3High and Tim-3Low populations were gated to analyze the relationship between Tim-3 expression and IL-2, IL-22 and TNF-α responses of CD4+ T cells. Numbers in each of dot plots represent the percentages of IL-2-, IL-22-, or TNF-α-producing CD4+ T cells. (A), (C), and (E) are representative flow cytometric dot plots showing de novo and Mtb-specific cytokine responses of TNF-α, IL-2 ,and IL-22 in Tim-3HighCD4+ T cell and Tim-3LowCD4+ T cells from an individual with active TB disease. (G), (I), and (K) shows that similar responses of TNF-α, IL-2, and IL-22 were also observed in Tim-3HighCD4+ T cell and Tim-3LowCD4+ T cells from an individual with LTBI. (B) is pooled flow cytometric data from individuals with active TB disease (n = 9) show that the percentages of TNF-α+CD4+T cells are much higher in Tim-3HighCD4+T cells than those in Tim-3LowCD4+T cells. Similar pooled flow cytometric data in (D) and (F), respectively, show that Tim-3HighCD4+T cells contained much higher percentages of IL-2+CD4+T cells or IL-22+CD4+T cells than their Tim-3Low counterparts. (H), (J), and (L) are similar pooled data from 9 individuals with LTBI show that Tim-3HighCD4+T cells contained much higher percentages of TNF-α+CD4+T cells, IL-2+CD4+T cells , and IL-22+CD4+T cells than their Tim-3Low counterparts. Data shown are representative of at least three independent experiments. Error bars represent SD. *** p<0.001, * p<0.05. (PDF) [file ppat.1002984.s005.pdf]

**Figure S6**

Active TB

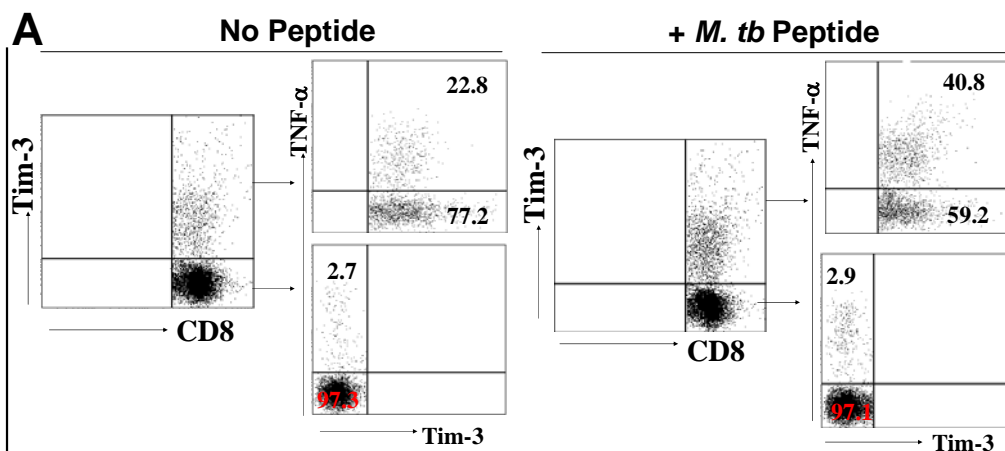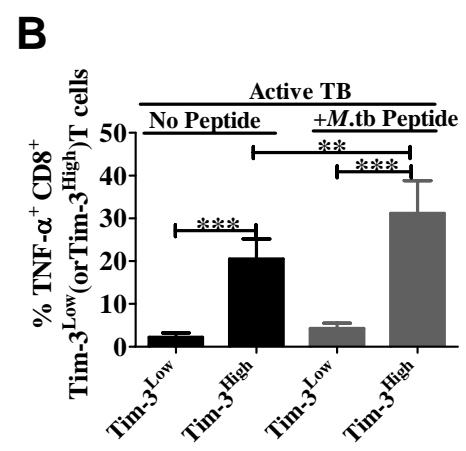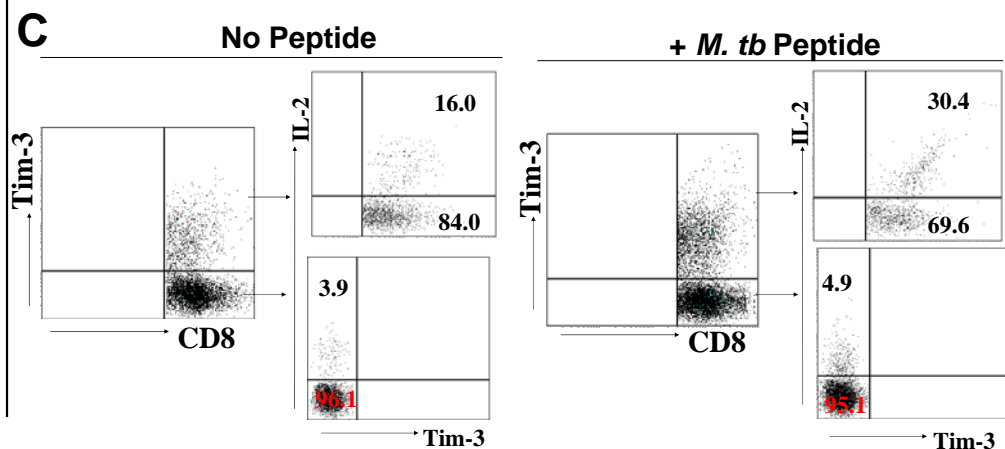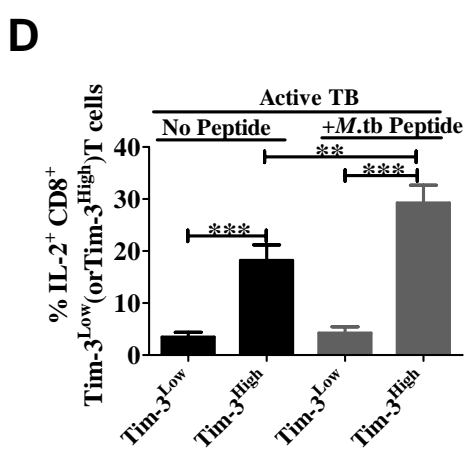

LTBI

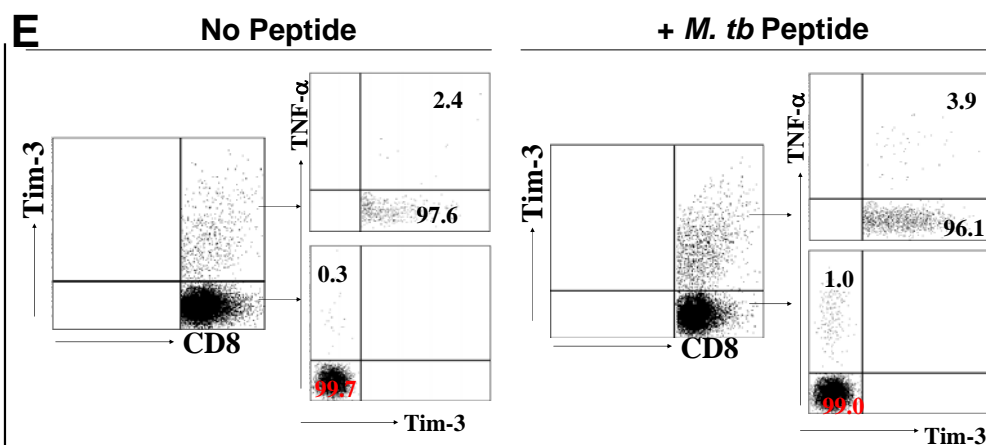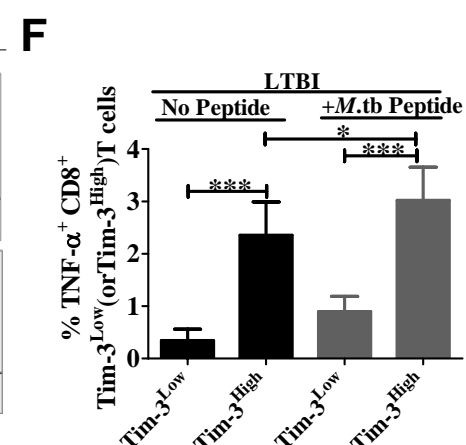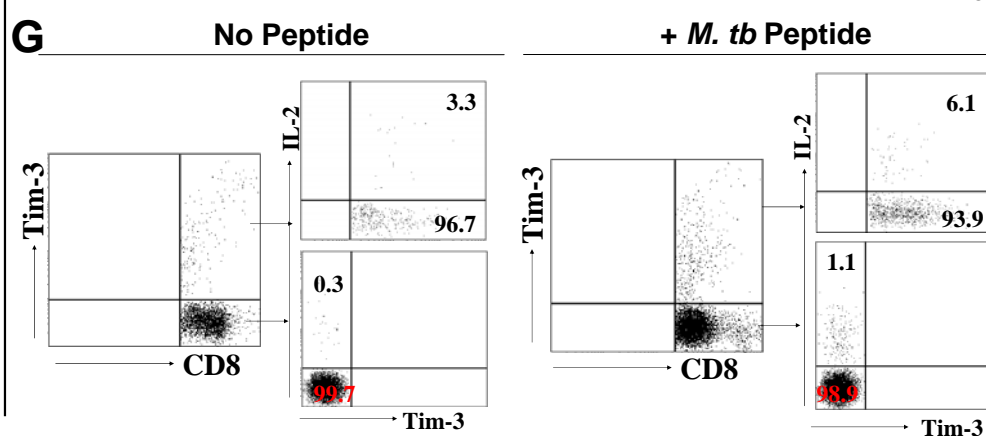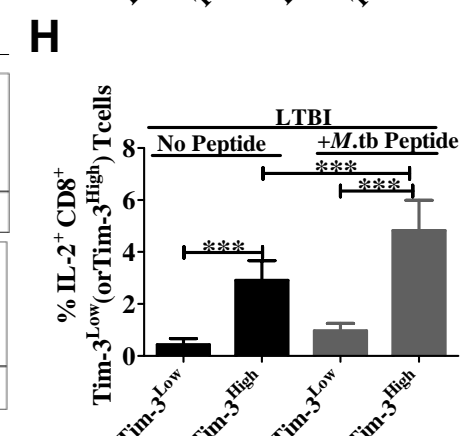

Supplement: Figure S6 — Tim-3High CD8+ T cells show stronger de novo or Mtb-specific responses of TNF-α and IL-2 than Tim-3LowCD8+ T cells. PBMCs derived from 9 individuals with untreated active TB disease or 9 individuals with LTBI were stimulated, stained, and analyzed using the same protocol as shown in Figure 2. Tim-3High and Tim-3Low populations were gated to analyze the relationship between Tim-3 expression and TNF-α and IL-2 responses of CD8+ T cells. Numbers in dot plots indicate the percentages of TNF-α- or IL-2-expressing CD8+ T cells. (A) and (C) are representative flow cytometric dot plots show de novo or Mtb-specific TNF-α and IL-2 responses in Tim-3HighCD8+ T cell and Tim-3LowCD8+ T cells from an individual with active TB disease. (E) and (G) are similar representative flow cytometric dot plots showing the similar de novo or Mtb-specific TNF-α and IL-2 responses in Tim-3HighCD8+ T cell and Tim-3LowCD8+ T cells from an individual with LTBI. (B) and (D) are pooled flow cytometric data from individuals with active TB disease showing that the percentages of either TNF-α+CD8+T cells or IL-2+CD8+ T cells are much higher in Tim-3HighCD8+T cells, as compared to Tim-3LowCD8+T cells (n = 9). (F) and (H) are similar pooled data from individuals with LTBI show that the percentages of either TNF-α+CD8+T cells or IL-2+CD8+ T cells are much higher in Tim-3HighCD8+T cells, as compared to Tim-3LowCD8+T cells (n = 9). Data shown are representative of at least three independent experiments. Error bars represent SD.* p<0.05, ** p<0.01, *** p<0.001. (PDF) [file ppat.1002984.s006.pdf]

**Figure S7****A****Active TB**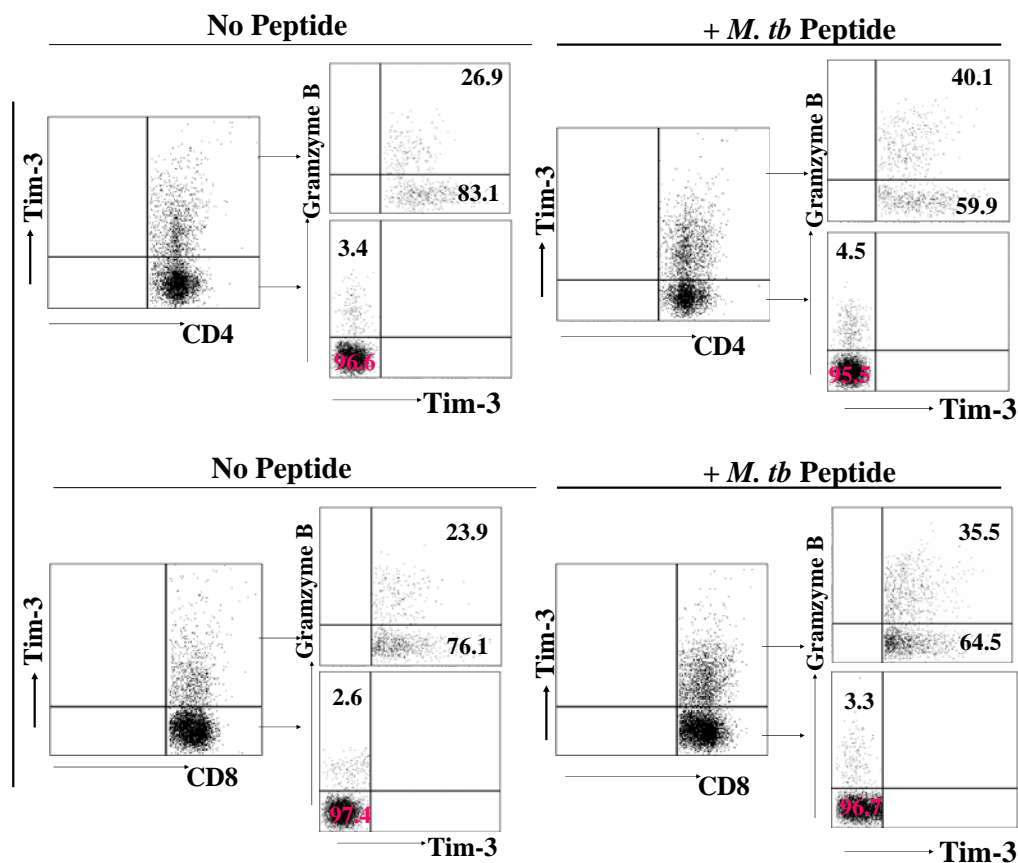**B**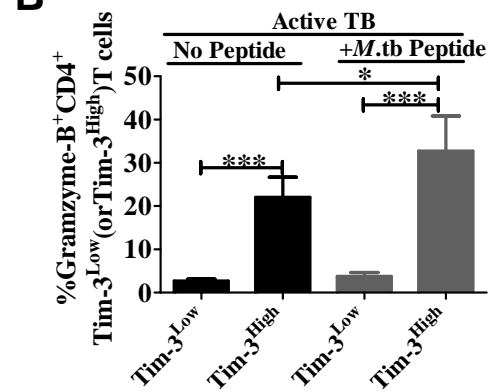**C**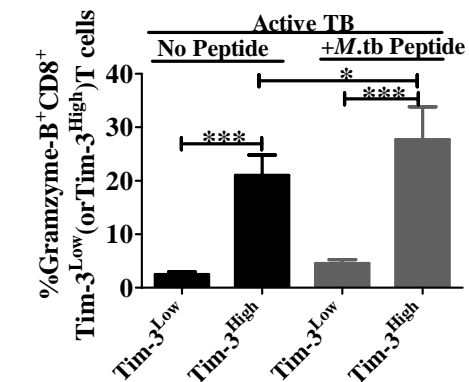**D****LTBI**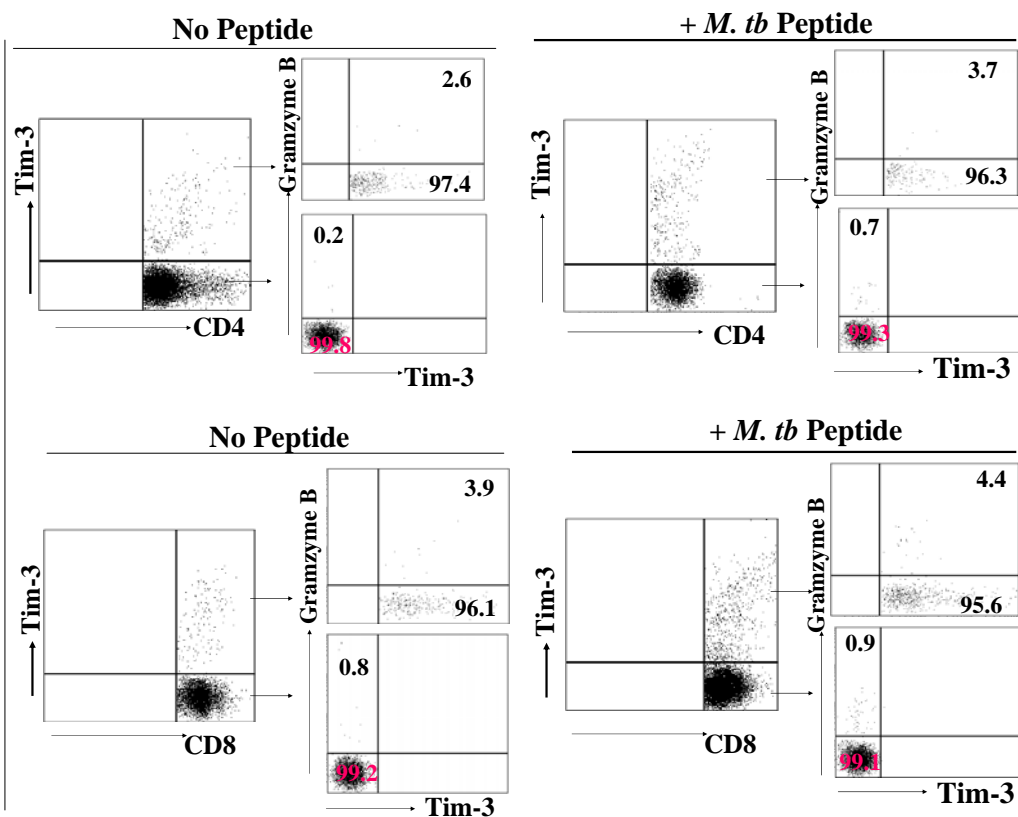**E**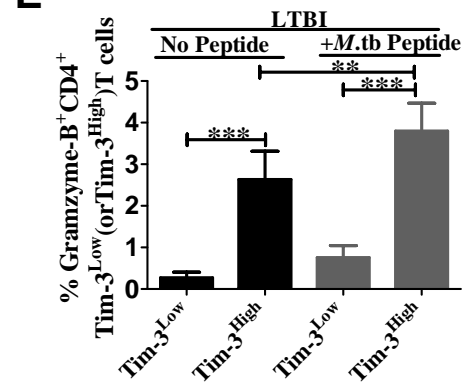**F**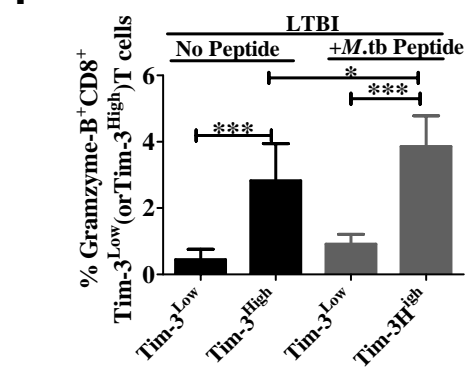

Supplement: Figure S7 — Tim-3 expression is associated with stronger granzyme B response of Mtb-specific CD4+ and CD8+ T cells. PBMCs derived from individuals with untreated active TB disease (n = 9) or with LTBI (n = 9) were cultured in presence or absence of pooled Ag85-b/ESAT-6 peptides, and analyzed as like perforin. (A) is representative flow cytometric dot plots showing the percentages of Mtb-specific or de novo production of granzyme with or without ex vivo stimulation of pooled Ag85-b/ESAT-6 peptides in CD4+ (or CD8+) T cells from an individual with untreated active TB disease. A two-tiered gating system was used as well to analyze Mtb-specific or de novo perforin production by Tim-3-expressing CD4+ (or CD8+) T cells, considering Tim-3High and Tim-3Low subpopulations. Numbers in each of dot plot represent the percentages of granzyme B-producing CD4+ or CD8+ T cells. (D) is similar flow cytometric dot plots that were shown to analyze granzyme B expression in CD4+ (or CD8+) T cells from an individual with LTBI. (B) and (C) are pooled flow cytometric data from individuals with active TB disease (n = 9) showing that the percentages of granzyme B+CD4+ (or CD8+) T cells are much higher in Tim-3HighCD4+ (or CD8+) T cells than their Tim-3Low counterparts. (E) and (F) are similar pooled flow cyotmetry data from individuals with LTBI (n = 9) showing that the percentages of granzyme B+CD4+ (or CD8+) T cells are much higher in Tim-3HighCD4+ (or CD8+) T cells than their Tim-3Low counterparts. Data shown are representative of at least three independent experiments. Error bars represent SD. * p<0.05, ** p<0.01, *** p<0.001. (PDF) [file ppat.1002984.s007.pdf]

**Figure S8**

**A**

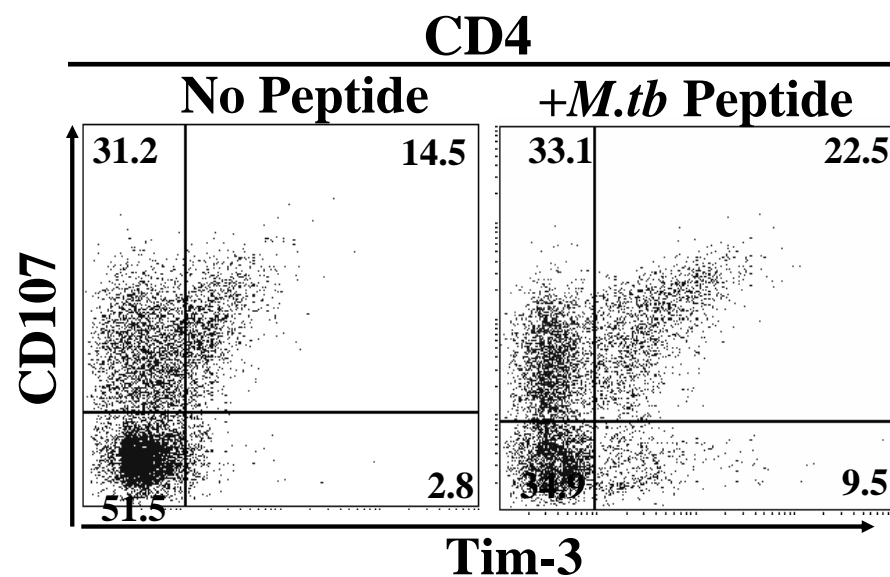

**B**

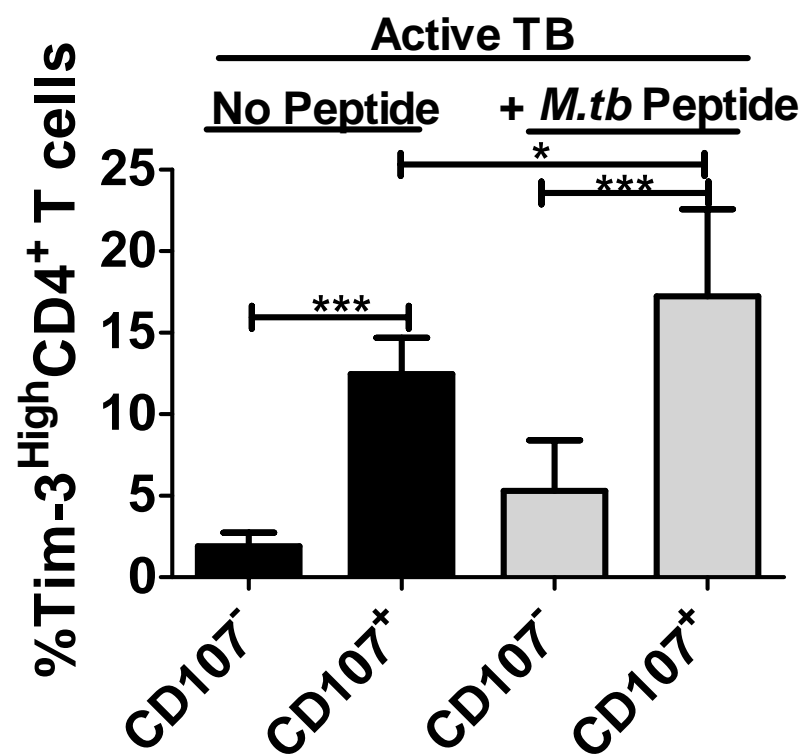

**C**

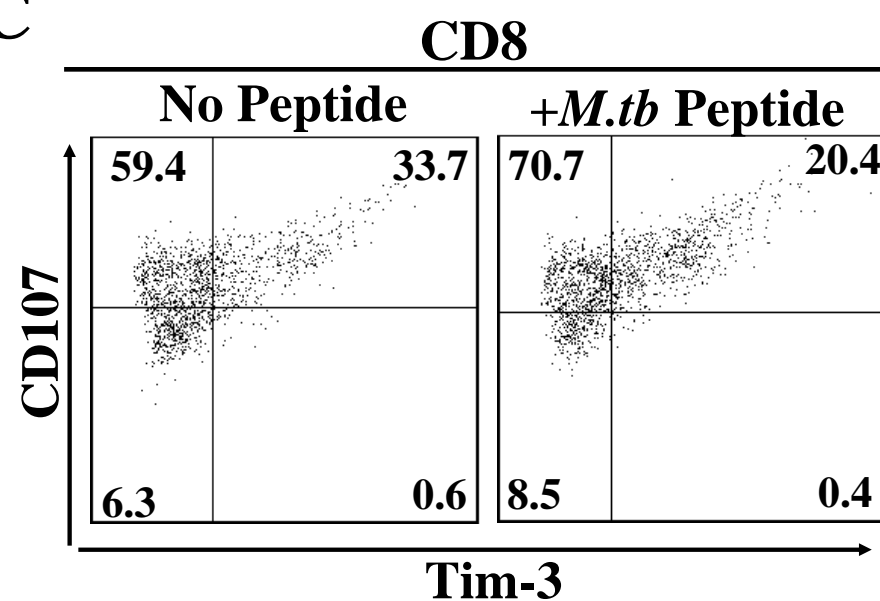

**D**

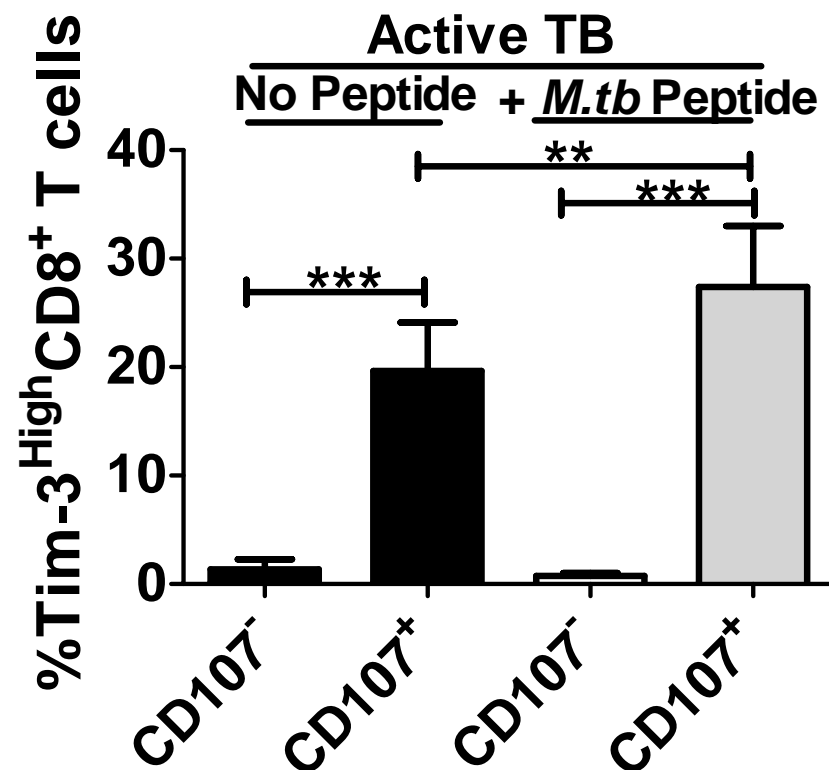

Supplement: Figure S8 — Tim-3 expression is associated with stronger degranulation capability of Mtb-specific CD4+ and CD8+ T cells. PBMCs derived from Mtb-infected individuals (n = 9) with untreated active TB disease were cultured in presence or absence of pooled Ag85-b/ESAT-6 peptides, and stained with fluorochrome-conjugated mAbs, followed by analysis using polychromatic flow cytometry. (A) is representative flow cytometric plots showing the percentages of CD107a expression with or without ex vivo stimulation of pooled Ag85-b/ESAT-6 peptides in Mtb-specific CD4+ and CD8+ T cells from an Mtb-infected individual with untreated active TB disease. (B) is complied flow cytometric data from Mtb-infected individuals (n = 9) showing that the percentages of Tim-3HighCD4+ T cells are much higher in CD107a+ T cells than those in CD107a− T cells. (C) and (D) are representative flow cytometric data and summary bar graphic data, respectively, showing that similar stronger CD107a expression as like Tim-3HighCD4+ T cells were also observed for Tim-3HighCD8+ T cells. Data shown are representative of at least three independent experiments. Error bars represent SD. * p<0.05, **p<0.01, *** p<0.001. (PDF) [file ppat.1002984.s008.pdf]

**Figure S9****A****Gated on CD3<sup>+</sup>CD4<sup>+</sup>T cells**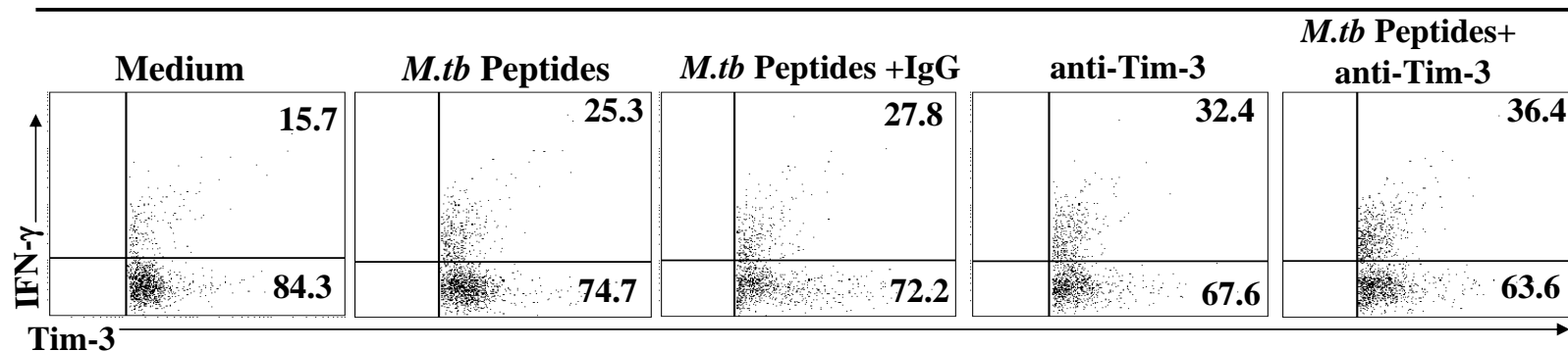**B****Gated on CD3<sup>+</sup>CD8<sup>+</sup>T cells**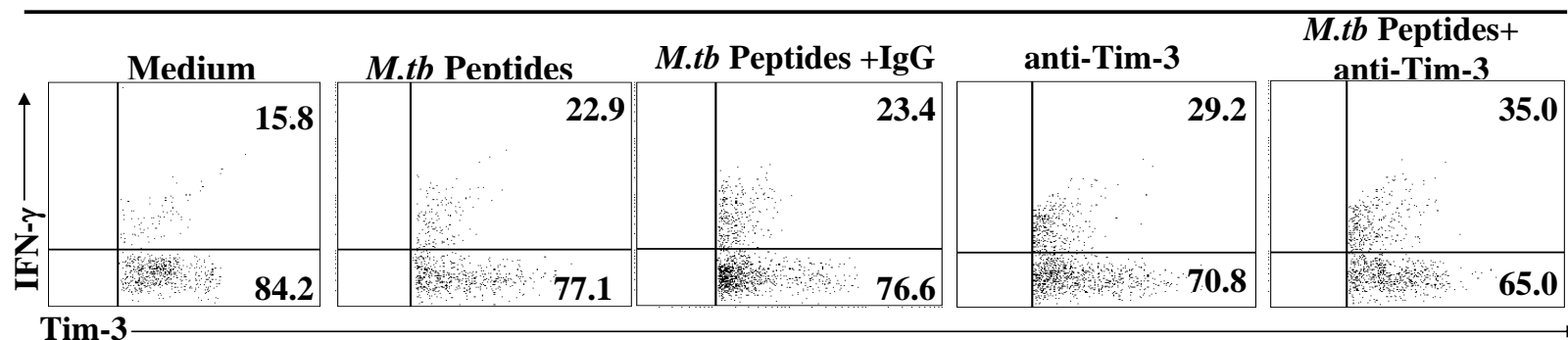**C**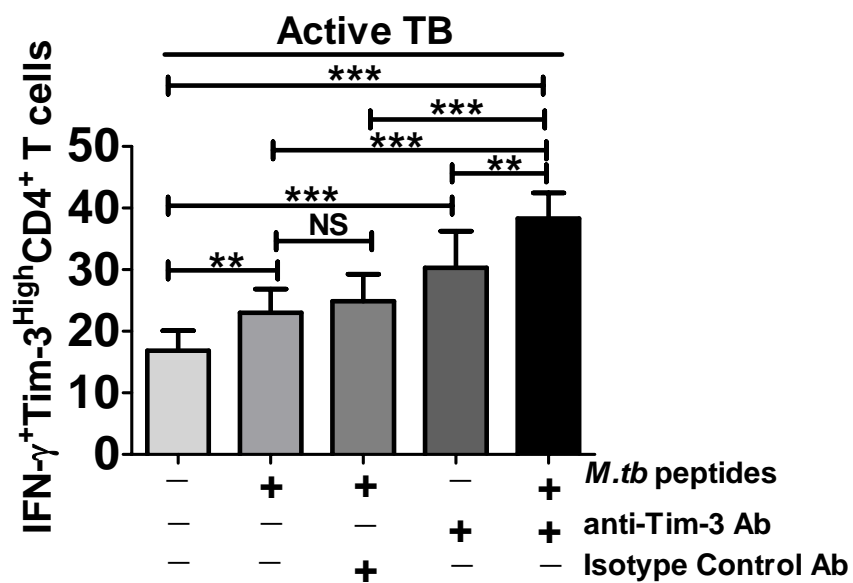**D**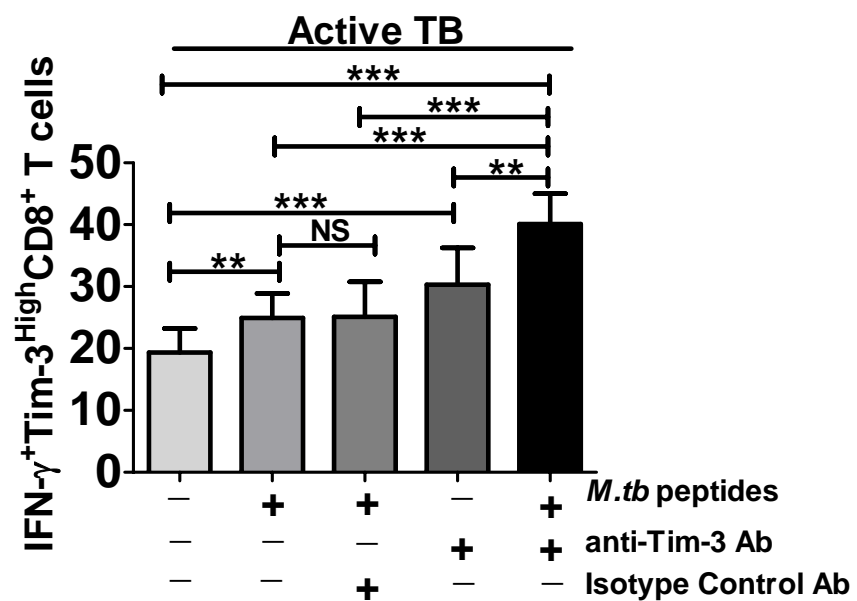

Supplement: Figure S9 — Stimulation of Tim-3 pathway using anti-Tim-3 Ab enhances the Mtb-specific effector functions of CD4+ and CD8+ T cells. PBMCs derived from Mtb-infected individuals (n = 9) with untreated active TB disease were stimulated ex vivo using anti-Tim-3 mAb (10 µg/ml) or isotype control Ab (10 µg/ml) in presence or absence of pooled Ag85-b/ESAT-6 peptides for 6 days. Cells were then stained using ICS protocol, and analyzed by flow cytometry. (A) is typical flow cytometric dot plots showing the effect of Tim-3 stimulation using anti-Tim-3 mAb on Mtb-specific IFN-γ response of Tim-3HighCD4+ T cells. Numbers in dot plots in each of sub-figure show the percentages of IFN-γ+Tim-3HighCD4+ T cells. (C) is summary bar graphic data showing that Tim-3 stimulation using anti-Tim-3 mAb but not isotype control Ab significantly enhances the production of Mtb-specific IFN-γ by Tim-3HighCD4+ T cells. (B) and (D) show that similar enhancement of Mtb-specific IFN-γ responses upon stimulation with anti-Tim-3 mAb (10 µg/ml) were also observed in Tim-3HighCD8+ T cells. Data shown are representative of at least three independent experiments. Error bars represent SD. ** p<0.01, *** p<0.001, NS, no statistical significance. (PDF) [file ppat.1002984.s009.pdf]
