# Supplementary material for: Tim-3-Expressing CD4+ and CD8+ T Cells in Human Tuberculosis (TB) Exhibit Polarized Effector Memory Phenotypes and Stronger Anti-TB Effector Functions
Source: PLoS Pathog. 2012 Nov 8;8(11):e1002984. doi: 10.1371/journal.ppat.1002984 (PMC3493466; doi:10.1371/journal.ppat.1002984)
Supplement: Table S1 — Clinical characteristics of the enrolled subjects. (PDF) [file ppat.1002984.s010.pdf]

Table S1

Group	HC	LTBI	Active TB
Cases	9	30	30
Sex (Male)	4 (45%)	17 (57%)	18 (60%)
Age (Years)	31±1.8	35±2.4	34±1.5
BCG-vaccinated	9 (100%)	30 (100%)	30 (100%)
HIV^+	0	0	0
TST^+	0	30 (100%)	30(100%)
AFB^+	0	0	25(83.3%)
$Culture^+$	0	0	30(100%)
PCR^+	0	0	30(100%)
Pulmonary TB	0	0	30(100%)
